# A systematic scoping review of mentor training in medical education between 2000 and 2024

**DOI:** 10.1186/s12909-025-07353-x

**Published:** 2025-07-24

**Authors:** Jun Rey Leong, Adele Yi Dawn Lim, Nila Ravindran, Darius Wei Jun Wan, Varsha Rajalingam, Jun Kiat Lua, Hannah Yi Fang Kwok, Krish Sheri, Victoria Jia En Fam, Ranitha Govindasamy, Nur Amira Binte Abdul Hamid, Michael Dunn, Lalit Kumar Radha Krishna

**Affiliations:** 1 Yong Loo Lin School of Medicine, National University of Singapore, NUHS Tower Block, Level 11, Block 1E, Kent Ridge Road, Singapore, 119228 Singapore; 2 Division of Supportive and Palliative Care, National Cancer Centre Singapore, 30 Hospital Boulevard, Singapore, 168583 Singapore; 3 Lee Kong Chian School of Medicine, Nanyang Technological University, 11 Mandalay Road, Singapore, 308207 Singapore; 4 Division of Cancer Education, National Cancer Centre Singapore, 30 Hospital Boulevard, Singapore, 168583 Singapore; 5 Division of Psychosocial Oncology, National Cancer Centre Singapore, 30 Hospital Boulevard, Singapore, 168583 Singapore; 6 Duke-NUS Medical School, National University of Singapore, 8 College Road, Singapore, 169857 Singapore; 7 Centre for Biomedical Ethics, National University of Singapore, Block MD11, 10 Medical Drive, #02-03, Singapore, 117597 Singapore; 8 Palliative Care Institute Liverpool, Academic Palliative & End of Life Care Centre, Cancer Research Centre, University of Liverpool, 200 London Road, Liverpool, Liverpool, L3 9TA UK; 9 Health Data Science, University of Liverpool, Whelan Building, The Quadrangle, Brownlow Hill, Liverpool , Liverpool, L69 3GB UK; 10 PalC, The Palliative Care Centre for Excellence in Research and Education, Dover Park Hospice, 10 Jalan Tan Tock Seng, Singapore, 308436 Singapore

**Keywords:** Mentor training, Medicine, Mentoring, Medical schools, Professional identity formation, Mentor, Mentee

## Abstract

**Background:**

Effective mentoring in medical education facilitates professional development amongst mentees and mentors, improves patient care and outcomes, as well as advances the reputation of the host organisation. Much of this success is guided, assessed and overseen by the mentor. Yet, mentor training remains inconsistent, poorly supported and often inadequately evaluated. Acknowledging mentor training as an essential aspect of mentoring programs, we propose a review to map current features and approaches to mentor training with the hopes of boosting the effective design of a proposed mentoring program.

**Methods:**

PubMed, Scopus, Embase, PsycINFO and CINAHL database searches were conducted for articles published between 1^st^ January 2000 and 31^st^ March 2024 on mentor training programs in medical education. This systematic scoping review was directed by a PRISMA-guided Systematic Evidence Based Approach (SSR in SEBA).

**Results:**

A total of 1124 abstracts were reviewed, 63 full-text articles were appraised and 69 articles were included. Five key domains were identified: 1) mentor qualities, 2) training structure, 3) content, 4) outcomes, and 5) obstacles.

**Conclusion:**

This SSR in SEBA reiterates the critical role of mentor training and introduces evidence of its impact on the professional identity formation (PIF) of prospective mentors. It also highlights that more programs are employing longitudinal mentoring processes to guide the inculcation of desired mentoring characteristics amongst prospective mentors. These efforts to nurture the PIF of the prospective mentor—to better shape their future mentee’s PIF—is novel and requires careful study.

**Supplementary Information:**

The online version contains supplementary material available at 10.1186/s12909-025-07353-x.

## Background

Effective mentoring in medical education facilitates professional development amongst mentees and mentors, improves patient care and outcomes and advances the goals of the host institution [1–3]. Recent studies have also demonstrated that the benefits extend to the personal sphere, with mentees and mentors alike reporting improved mental well-being through the provision of psycho-emotional support, a sense of accomplishment and a sense of purpose [1–3].

In order to achieve the goals and reap the benefits of mentoring, the mentor plays a critical role [4]. Krishna and Renganathan [5] report that mentors provide intentional, longitudinal, personal and professional role modelling; clinically and contextually-sensitive tutoring and supervision; assessment-led coaching; remediation; and mentoring (henceforth referred to as the *mentoring umbrella*). In addition, Sheri et al. [6] noted that mentors must be able to nurture personalised, open, trusting and enduring mentoring relationships, along with sustaining a supportive and open mentoring environment [6]. More recently, data have suggested that mentors also play a pivotal role in the socialisation process, imbuing mentees and peer mentors with the program’s values, beliefs, principles and shared identity [7–10]. Building upon Sheri et al. [6]’s work, a review of regnant data on mentor training is thus proposed to better appreciate the role of mentor training programs and their impact on the professional identity of mentors, as mentoring programs are increasingly integrated into curricula.

This systematic scoping review (SSR) thus aims to answer the primary research question,* “What is known about mentor training in medical education?”*, and secondary research questions, *“What are the key features of mentor training?”* and *“How are mentor training programs designed, structured, implemented and evaluated?”*

## Methods

### Theoretical lens

We employed a SSR guided by the Systematic Evidence-Based Approach (SEBA) [2, 11–14] to explore data on mentor training in medical education. This review was intended to build on Sheri et al.’s [6] review by expanding the understanding of mentoring to include all aspects of the *mentoring umbrella* to nurture mentors through mentor training programs [15, 16]. This also required careful consideration of both individual and contextual factors that influence the mentoring process (Table 1). As part of a reflexive approach, the research team continuously reviewed the data individually and collectively through online and in-person discussions to ensure that the data sufficiently captured these considerations.

**Table 1:** Individualised and contextual considerations shaping mentoring experiences

*Individual Considerations*	*Contextual Considerations*
• Belief systems and personal values • Motivations and willingness to engage in the learning and meaning-making processes; and the ability to discern and balance relevant considerations according to their impact • Background, such as preferred working styles, opportunities [17–22], experience, skills and goals, as well as demographic [17, 18] and socio-cultural [19–21] features • Psycho-emotional well-being and the adoption of reflective practice [23–25], along with personal coping strategies [2, 26–50]	• The mentoring program’s setting in a formal or informal curriculum [51–55]; and attention to professional identity formation (PIF) [56–58], hidden curriculum [39, 57, 59, 60, 67], prevailing discourses [37, 63, 68–70], daily activities [61, 71, 72], and rites of passage [39, 66, 73–79] • Organisational factors, such as administrative support [80], faculty training and evaluation [80, 81] • Practice differences across different training sites, including case mix and workload, which may evolve over time [57, 82–85] • The program’s learning objectives [86], goals [87, 88], timelines and professional standards [89, 90], codes of conduct, expectations [91–93], sociocultural norms and legal requirements [94–98], which are embedded into competency-based mentoring stages • Practice culture shaped by the program’s hidden curriculum [39, 57, 59–67] • Faculty selection and characteristics, including personality, working style, competencies and commitment to mentoring [57, 82, 84, 85, 99–125] • Accessible communication [110, 119–121, 126] and timely, longitudinal training support that caters to the physician’s personal needs [112, 127, 128] and changing contextual considerations [82, 83, 100, 101, 104, 106, 110–112, 116, 117, 120, 123, 125, 129, 130]

### Stage 1 of SEBA: systematic approach

**Fig. 1:**
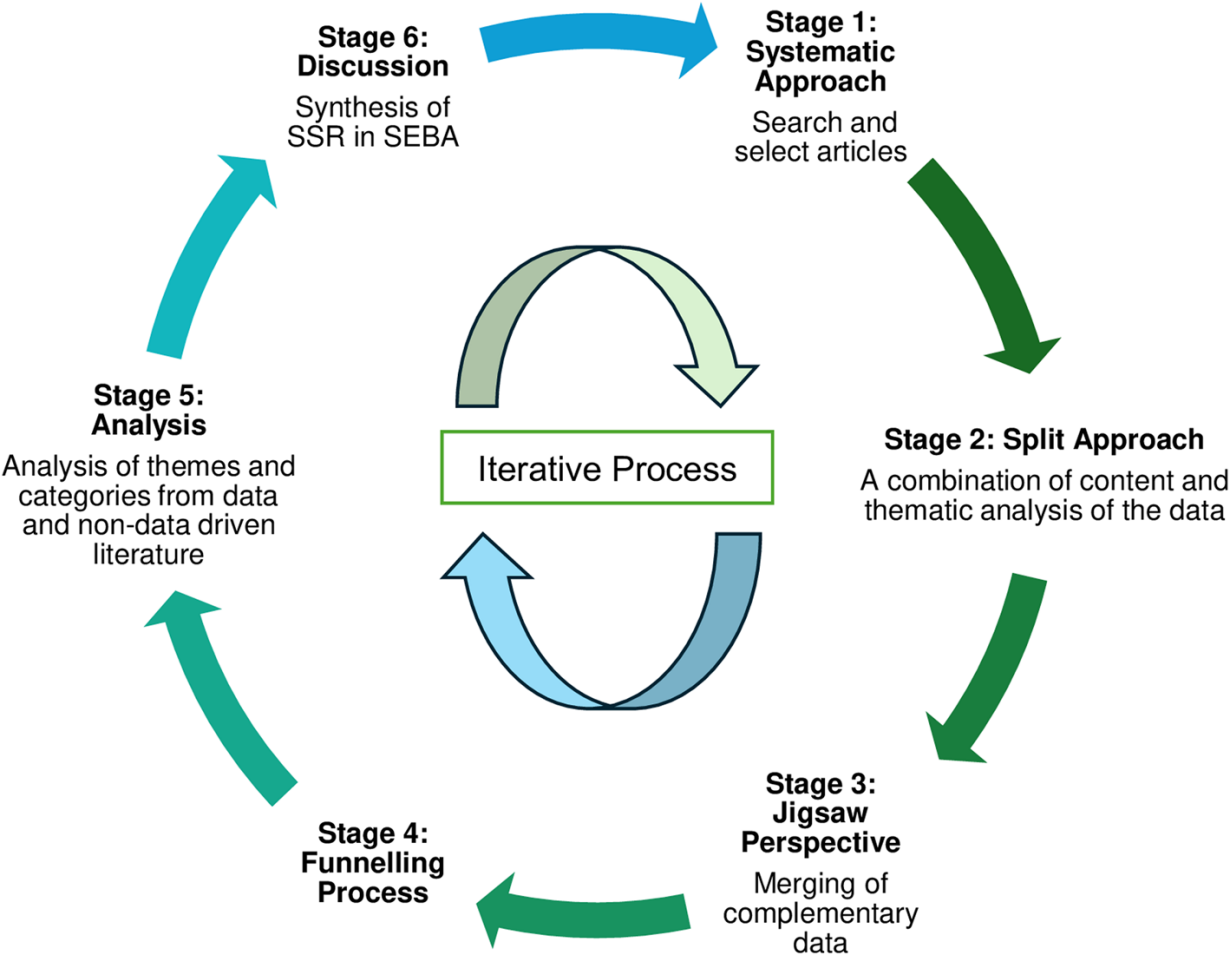
The Systematic Evidenced-Based Approach (SEBA) process

To bolster reproducibility and accountability, the review was guided and supported by an expert team that oversaw the stages of SEBA. This team of experts included medical librarians from the Yong Loo Lin School of Medicine (YLLSoM), along with local educational experts and clinical practitioners at YLLSoM, the National Cancer Centre Singapore, the Palliative Care Institute Liverpool and Duke-NUS Medical School. The six stages of SEBA are outlined in Fig. 1.

#### Determining the title, research question(s) and inclusion criteria

To address the primary research question,* “What is known about mentor training in medical education?”* and secondary research questions, *“What are the key features of mentor training?”* and *“How are mentor training programs designed, structured, implemented and evaluated?”*, the Population, Intervention, Comparison, Outcome, Study Design (PICOS) framework was adopted (Table 2) in compliance with the PRISMA-ScR guidelines (Additional File 1).

**Table 2:** Population, Intervention, Comparison, Outcome and Study Design (PICOS) framework and inclusion and exclusion criteria applied to database search

PICOS	Inclusion Criteria	Exclusion Criteria
Population	• Medical students and physicians at all stages of training	• Non-medical specialties, such as veterinary and dentistry • Allied health professionals • Programs using methods of instructions such as supervision, advisorship, preceptorship, role modelling, coaching and teaching alone without mentoring
Intervention	• Formal and informal mentor-training programs for mentor preparation in all forms • Preparation for new mentors or refresher courses for current mentors • Mentor-training programs or methods in hospital-based or medical school settings, or clinical and translational science	
Comparison	• Comparisons of mentoring training programs or their assessments	
Outcome	Outcomes of mentor-training on the mentor, mentee or organisation	
Study Design	• Articles in English or translated to English • All study designs and article types • Year of Publication: 1^st^ January 2000 - 31^st^ March 2024 • Databases: PubMed, Embase, PsycINFO, Scopus, CINAHL	Non-English language articles

#### Searching

Between 12^th^ September 2023 and 4^th^ April 2024, the research team conducted independent searches of articles on mentor training published between 1^st^ January 2000 and 31^st^ March 2024 featured in PubMed, Scopus, Embase, PsycINFO and CINAHL databases. To further enhance the review, ‘snowballing’ of references from the included articles was performed. This saw the inclusion and re-evaluation of all included articles featured in Sheri et al.’s [6] review. The full search strategy is enclosed in Additional File 2.

#### Extracting and charting

The research team independently reviewed the abstracts using an abstract screening tool, reaching consensus on the final list of articles to be included through ‘negotiated consensual validation’ [131]. This practice of collaborative discussion encouraged a healthy negotiation of points of agreement and disagreement to attain unanimity within the team.

### Stage 2 of SEBA: split approach

To capture a more holistic and refined analysis of the data, this stage involved the simultaneous application of thematic and directed content analysis of the included full-text articles. This approach offered a means to circumnavigate the shortcomings of each method of data analysis, such as the lack of depth in directed content analysis and the subjectivity in thematic analysis. This stage was divided among three teams as follows.

#### Tabulated summaries

The first team of researchers summarised and tabulated the included articles, in keeping with Wong et al.’s [132] *“Realist and Meta-narrative Evidence Syntheses—Evolving Standards (RAMESES) Publication Standards”* and Popay et al.’s [133] *“Guidance on the Conduct of Narrative Synthesis in Systematic Reviews”.* This ensured that the fundamental details of each article were captured. The tabulated summaries are detailed in Additional File 3.

#### Thematic analysis

Guided by Braun and Clarke’s [134] approach to thematic analysis, the second team studied the included articles to identify key meanings and patterns. Subsequently, they crafted ‘codes’ from the surface meanings of the text, organising them in a code book for further step-by-step analysis. This iterative process saw the merging of new codes with previous ones. As an inductive approach, new themes were elicited *“from the raw data without any pre-determined classification”* [135]. The independent findings were discussed in team meetings where the final list of themes was concluded through ‘negotiated consensual validation’ [131].

#### Directed content analysis

Simultaneously, Hsieh and Shannon’s directed content analysis [136] was applied by the third team to highlight and operationalise a priori coding categories from Sheri et al.’s [6] *“A Framework for Mentor Training Programs: A Scoping Review of Mentor Training Programs in Medicine between 1990 and 2017”*. New codes were assigned to any data that did not match existing ones. Agreement on the final list of categories was similarly reached through ‘negotiated consensual validation’ [131].

### Stage 3 of SEBA: Jigsaw perspective

Likened to pieces of a jigsaw puzzle, the research team merged overlapping and complementary findings from both methods to form broader puzzle pieces, known as themes/categories [137].

### Stage 4 of SEBA: funnelling process

The themes/categories were subsequently compared with the tabulated summaries to ensure that vital information was retained and omissions were minimised [138]. This led to the formation of key domains that formed the basis of the ensuing discussion.

### Stage 5: analysis of evidence-based and non-data-driven literature

To assuage concerns regarding the plausibility of bias from non-data-based articles (i.e., grey literature, opinion, perspectives, editorial and letters), the themes and categories from the aforementioned sources were compared with that of evidence-based publications. The themes and categories in both groups were found to be similar, that is, the non-data-based articles did not bias the analysis.

## Results

**Fig. 2:**
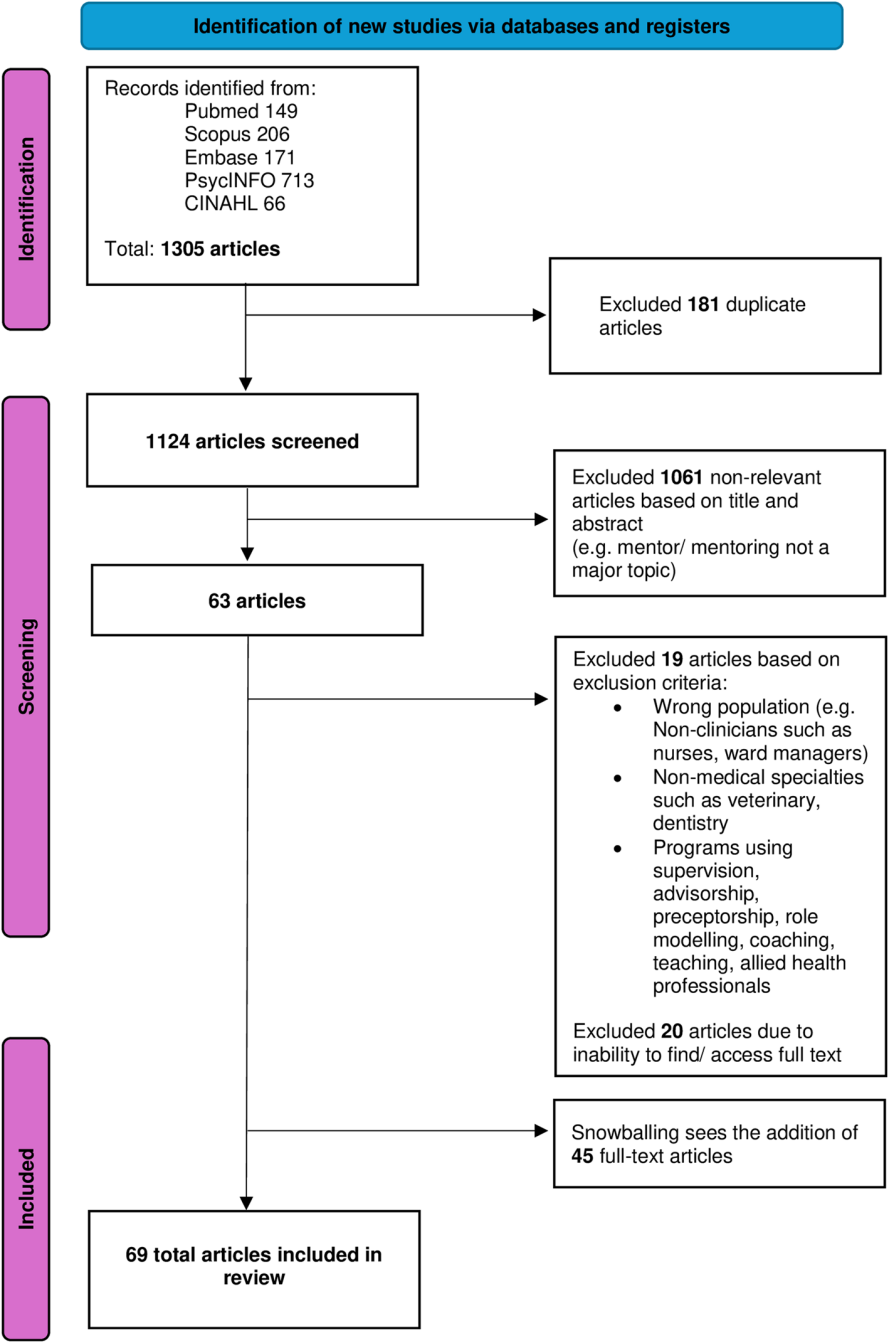
PRISMA flowchart

A total of 1124 abstracts were reviewed, 63 full-text articles were appraised and 69 articles were included (Fig. 2). The included articles revealed 5 key domains: 1) mentor qualities; 2) content; 3) training structure; 4) outcomes; and 5) obstacles.

### Domain 1. Mentor qualities

The ideal mentor ought to be supportive and trusting on a personal front, sensitive to diversity-related issues as a communicator and possess integrity and passion for teaching as an educator, replete with qualifications and experience as a professional. Both personal and professional qualities are necessary to enhance mentoring success by facilitating open and trusting mentoring relationships. Further details of ideal mentor qualities can be found in Table 3.

**Table 3:** Ideal mentor qualities

**Sub-Domain**	**Mentor Qualities**	**References**
**Personal**	Demonstrates virtues such as kindness, dedication, humility and integrity	[6, 139–164]
	Reflects on practice and teaching	[6, 140, 155, 161, 165]
	Compatible traits with mentee, leading to trust between mentor–mentee	[143, 153, 157, 160, 166]
	Curious and has fervor for learning	[140, 149]
	Approachable and accessible to learners	[140, 143, 146, 150, 156, 160, 167]
**Professional**	Interest in subject and passion for teaching	[6, 140–142, 145, 147, 151, 152, 156, 162, 168–173]
	Good communicator, including in the use of technology, and sets goals and expectations	[6, 140–143, 145–149, 151, 152, 154–162, 166, 168, 170, 174, 175, 178, 179]
	Responsive and adaptive, able to personalise mentoring	[6, 139, 140, 142, 143, 145, 146, 148, 152, 156, 158, 162, 170, 176, 177, 179, 180]
	Role model and positive influence	[6, 140–142, 145–147, 155, 160, 162, 166, 177, 179, 181]
	Effective supervision and teaching	[6, 141, 142, 144–146, 148, 154, 156, 158, 168, 170, 181–184]
	Qualified and experienced	[6, 140, 142, 143, 146, 147, 150–152, 155, 162, 164, 168–171, 173]
	Maintains patient safety	[140, 142, 146, 181]
	Sensitive to diversity-related issues	[148, 152, 153, 161, 163, 176, 185]
	Partakes in quality improvement	[140, 142, 146, 147, 162, 165, 170, 172, 173]

### Domain 2. Content

Similarly, the contents of mentor training are varied and listed below in Table 4. Of note is the general focus on leadership, teaching, nurturing mentoring relationships and promoting professional communication.

**Table 4:** Contents of mentor training

Sub-Domain	Features	References
Interpersonal skills	Communication skills, such as active listening and building relationships	[6, 141, 145, 147, 148, 151, 152, 155, 157, 158, 160, 161, 164, 167–169, 174, 176–178, 182, 184, 186, 187, 183–192]
	Emotional intelligence	[145, 155, 171, 186, 191]
	Management and conflict resolution	[6, 140, 146, 154, 160, 164, 167, 169, 186, 189, 191, 193]
	Team player and enhancing team dynamics	[141, 154, 186]
Nurturing of mentoring relationships	Aligning expectations	[6, 146, 147, 152, 158, 175–177, 184, 187–190, 194, 195]
	Sensitivity to diversity	[6, 145, 148, 151–153, 155, 158–161, 163, 171, 173, 176, 184, 189, 190, 196]
	Creation of supportive environment	[140, 145, 147, 149, 152, 155, 159, 160, 171, 173, 188, 189, 195]
	Sensitivity to mentees' needs	[140, 169, 171]
Skills	Leadership	[154, 155, 157, 168, 194, 197]
	Ethics	[6, 154, 160, 164, 186, 198]
	Networking	[144, 148, 153, 157, 158, 160, 164, 173, 178]
	Self-appraisal	[140–142, 146, 147, 152, 158, 160, 162, 168, 170, 173, 175, 177, 186–188, 195, 199]
	Work-life balance	[151, 154, 155, 176, 188, 193]
	Feedback	[6, 139, 140, 142, 146, 149, 150, 154, 157, 160, 173, 175–179, 181, 184]
	Reflective cycles and debriefs	[6, 139, 140, 142, 146, 149, 150, 154, 157, 173, 175–179, 181, 184]
	Role modelling	[6, 141, 142, 153, 160, 169, 172, 174, 175, 186]
	Teaching • Content delivery • Independence • Resourcefulness	[6, 140, 151, 167, 170, 175, 181, 187, 194, 197, 200, 201] [148, 149, 155, 161, 164, 176, 187–190] [6, 140, 154, 155, 169, 173, 197]
	Mentoring	[6, 139, 186]
Oversight	Overseeing compliance and progress	[6, 139, 140, 145, 147, 148, 151, 153–155, 158, 161, 162, 164, 168, 175, 176, 178, 180, 184–191, 195, 197]

### Domain 3. Training structure

Whilst variations in the learning objectives, stages of mentor training, frequency, duration and format of mentor training sessions are evident, some common threads are discernible.

First, learning objectives are focused upon inculcating the desired characteristics and developing mentoring skills and humanistic practitioners [139, 141, 156, 168, 170, 172, 175, 182, 184, 186, 202]. On a macro-level, this enables healthcare institutions to scale up training capacity and meet healthcare goals [168, 174, 182].

Second, mentor training takes on three key stages:

- To start, the **pre-training** stage seeks to prepare mentors for their anticipated roles, tasks and responsibilities; align expectations; match new mentors to senior mentors; and create a nurturing mentoring environment [6, 139, 140, 142, 145, 146, 168, 174, 176, 184, 186, 196, 198, 200–203].
- This is then proceeded by the training stage that aims to equip mentors with key competencies to support, assess and provide feedback, including effective communication skills [139, 142, 144, 145, 149, 176–178, 188, 200–202]. These sessions tend to be interactive, immersive and focused on refining skills [139–141, 144, 145, 154–156, 168–174, 176, 179, 182, 184, 186–191, 197, 198, 200, 202–205]. Mentors are also equipped with self-appraisal and reflective skills to continue self-directed improvement in their mentoring abilities [156, 186].
- Finally, post-training focuses on furthering mentor development by involving them in the recruitment of new mentors and in the design and refinement of training programs [139, 141, 156, 157, 168, 169, 172, 174, 182, 191, 197, 198, 200, 204].

Third, mentor training sessions take on various modalities, ranging from the common workshops and didactic presentations, to the less frequently adopted role plays, teleconferences and video-based discussions (Table 5).

**Table 5:** Training modalities

Mode of delivery	Count	References
Workshop	22	[6, 139, 145, 152, 154, 155, 166, 174, 176–178, 182, 184, 185, 187, 191, 192, 195, 198, 203, 205, 206]
Small group activities and discussions	20	[139, 141, 152–154, 156, 167, 168, 173, 176, 186, 188, 189, 191, 192, 198–201, 204]
Didactic presentation	16	[6, 139, 152, 154, 159, 167, 172, 173, 176, 177, 181, 191, 193, 201, 204, 205]
Seminar	15	[6, 144, 147, 149, 151, 166, 169, 171, 174, 177, 188, 191, 192, 201, 205]
Case-based discussion, which may involve the use of videos	11	[139, 141, 145, 147, 152, 156, 159, 191, 198, 199, 205]
Teleconference	9	[6, 139, 164, 170, 185, 187, 198, 200, 204]
Patient simulation and role-play	16	[6, 140–142, 149, 159, 162, 168, 172, 176, 179, 198, 200, 202, 204, 206]
Reflection session	7	[144, 153, 156, 176, 182, 186, 202]
Active learning curriculum	5	[162, 190, 197, 198, 204]

### Domain 4. Outcomes

Mentor training advances the professional and personal interests of the mentee and mentor while also boosting the host organisation’s reputation and ability to meet professional standards, ultimately leading to enhanced patient care and outcomes. We consider each in turn.

i. Mentee-related outcomes*:* Career development, improved learning, enhanced clinical competency and greater productivity on a professional level [6, 141, 143, 144, 147, 148, 151, 152, 157–160, 164–169, 171–173, 175–179, 184, 186–190, 193–195, 197, 199, 201] and improved mental well-being and self-esteem on a personal level [6, 141, 147, 156, 165–168, 171, 177, 188, 193].
ii. Mentor-related outcomes: Improved knowledge, skills and competencies as mentors and educators, and enhanced professional identity formation [6, 139, 141, 142, 144–148, 150–152, 155–160, 162, 164–166, 168–170, 173, 175, 176, 178, 179, 181, 182, 184, 186, 188–193, 195–199, 201–203, 206], alongside greater personal satisfaction and confidence in mentoring skills [141, 143, 146–148, 150, 151, 155, 160, 162, 165–168, 176, 177, 180, 187, 188, 195, 201].
iii. Organisational outcomes: Improvements in quality of care, productivity, work culture, staff retention and the upholding of clinical standards, leading to downstream benefits for patients [6, 141, 143, 147, 150, 152, 155, 156, 159, 160, 164–169, 171, 173, 176, 177, 179, 180, 186–190, 192, 197, 201, 202, 205].

### Domain 5. Obstacles

Mentor- and trainer-related obstacles include inadequate training and a lack of time and availability [139–141, 144, 147, 148, 151, 152, 154, 156, 157, 159, 162, 165, 166, 168–173, 175–179, 181, 182, 184, 187, 188, 190, 194, 197, 201, 203, 205] whilst a dearth of investment in resources and poor sustainability of facilities comprise organisation-related obstacles [6, 140–143, 146–148, 151, 152, 154, 156, 157, 159, 162, 165, 167, 169–171, 173, 176, 177, 179, 184, 187, 190, 192, 194, 195, 197, 201–203].

## Discussion

### Stage 6 of SEBA: synthesis of discussion

In answering our primary research question, *“What is known about mentor training in medical education?”*, this SSR in SEBA reveals that mentor training is acknowledged as a valuable aspect of the mentoring process. Whilst there exists much variety in mentor training programs, such as in content and delivery methods, this review, in addressing the secondary research question, identifies key features that remain consistent across training programs. For one, consistently desired characteristics of successful mentors are sought in mentor recruitment. Meanwhile, the content of mentor training programs is shaped by key mentoring issues and the need to support mentoring relationships, alongside changing individual and organisational considerations*.*

More recent reviews highlighting mentor training objectives—focused on personal and professional development, building humanistic features and practicing professional and interprofessional skills—serve to underscore efforts to shape the PIF of mentors. For example, incorporating recruitment efforts and having prospective mentors build training capacity and advance organisational and program goals help imbue them with the desired belief systems and shared identity that they must identify and nurture amongst their mentees and peers.

These wider perspectives of mentor training underline the structured, stage-based approach increasingly featured in newer longitudinal training programs. Over time, mentors practice role modelling of requisite skills and attitudes; demonstrate teaching and tutoring skills in instilling knowledge; and support the continuous use of formal and informal assessments that direct timely and effective blending of elements within the *mentoring umbrella*. This longitudinal investment extends beyond providing prospective mentors with lived experiences of their ‘craft’ and towards greater identification with their roles and responsibilities. To this end, portfolios that allow the compilation of multi-modal, multi-rater assessments, feedback and reflections may be a suitable evaluation tool to map the self-development journeys of mentors [207–210]. Furthermore, reflections not only capture the depth of the experience in the mentoring program, but also serve as catalysts for introspection, thereby improving one’s capacity for reflective practice and mentorship [211–213].

Beyond drawing on elements of the Adult Learning Theory [214, 215], a longitudinal mentored training program also highlights the role of the host organisation. In overseeing the design, resourcing, structuring and support of this longitudinal, structured, stage-based training program, the host organisation is responsible for nurturing ‘fit-for-purpose’ mentoring relationships which attend to changing competencies, availabilities, interests and goals over the mentoring journey. Similar ‘fit-for-purpose’ mentoring relationships between mentors and their mentees have been shown to be integral in effective mentoring outcomes. Only through regular assessment and feedback channels and investment in resources and manpower for mentor training programs can host organisations fully reap the benefits of mentoring, ultimately improving patient care.

### Limitations

This review combines data from medical students, residents and senior clinicians. While providing unique perspectives, these backgrounds differ significantly in their qualifications and experience, thus influencing the requirements and objectives to facilitate and achieve effective mentorship. Additionally, the focus on largely North American and European practices, the inclusion of only English-language articles and the defined date range collectively limit the generalisability of our findings to other non-Western settings, given the sociocultural nuances of medical practice and mentoring culture. However, despite these limitations, considering the common ethos of medical practice to uphold patient welfare and safeguard ethical principle, it is believed that the results of this review may still be applicable across all contexts, though host organisations are advised to adapt the findings to their local practice settings and needs.

## Conclusion

This review has identified the qualities of an ideal mentor, the content and pedagogy of training programs, outcomes and barriers. In doing so, it is proposed that mentor training should focus not only on knowledge and skills, but also the desired belief systems and shared identities of mentors. Both the personal and professional development of mentors should be invested by the host organisation in order to reap longitudinal gains.

## Supplementary Information

Additional file 1. PRISMA-ScR Checklist.

Additional file 2. Full Search Strategy.

Additional file 3. Tabulated Summary of Included Articles.

## Data Availability

The datasets supporting the conclusions of this article are included within the article and its additional files.

## Abbreviations

SSR: Systematic Scoping Review
SEBA: Systematic Evidence-Based Approach
PIF: Professional Identity Formation
PICOS: Population, Intervention, Comparison, Outcome, Study Design
