# Supplementary material for: A systematic scoping review of mentor training in medical education between 2000 and 2024
Source: BMC Med Educ. 2025 Jul 24;25:1110. doi: 10.1186/s12909-025-07353-x (PMC12291282; doi:10.1186/s12909-025-07353-x)
Supplement: Supplementary file 2 — Additional file 2. Full Search Strategy. [file 12909_2025_7353_MOESM2_ESM.docx]

**Additional File 2. Full Search Strategy**

**PubMed (last searched on 26/3/22)**

*Publication date: 1/1/18-31/12/22

| #1 | “students, medical” [MeSH Terms] OR “medical student” [Title/Abstract] OR “medical students” [Title/Abstract] OR “medical undergraduate students” [Title/Abstract] OR “medical undergraduates students” [Title/Abstract] OR “medical postgraduates” [Title/Abstract] OR “medical postgraduates” [Title/Abstract] OR “physicians” [MeSH Terms] OR “physician” [Title/Abstract] OR “physicians” [Title/Abstract] OR “doctor” [Title/Abstract] OR “doctors” [Title/Abstract] OR “clinicians” [Title/Abstract] OR “resident” [Title/Abstract] OR “residents” [Title/Abstract] OR “medical practitioner” [Title/Abstract] OR “medical practitioners” [Title/Abstract] OR “consultants” [Title/Abstract] OR “consultant” [Title/Abstract] OR “attendings” [Title/Abstract] OR “attending” [Title/Abstract]  ("students, medical"[MeSH Terms] OR "medical student"[Title/Abstract] OR "medical students"[Title/Abstract] OR "medical undergraduate students"[Title/Abstract] OR "medical postgraduate"[Title/Abstract] OR "medical postgraduates"[Title/Abstract] OR "physicians"[MeSH Terms] OR "physician"[Title/Abstract] OR "physicians"[Title/Abstract] OR "doctor"[Title/Abstract] OR "doctors"[Title/Abstract] OR "clinician"[Title/Abstract] OR "clinicians"[Title/Abstract] OR "resident"[Title/Abstract] OR "residents"[Title/Abstract] OR "medical practitioner"[Title/Abstract] OR "medical practitioners"[Title/Abstract] OR "consultants"[Title/Abstract] OR "consultant"[Title/Abstract] OR "attendings"[Title/Abstract] OR "attending"[Title/Abstract]) AND ((2018/1/1:2022/12/31[pdat]) AND (english[Filter])) | 341, 242  Student  Students  studenting  student*  344,870 |
| --- | --- | --- |
| #2 | "mentoring" [MeSH Terms] OR "novice mentor" [Title/Abstract] OR "undergraduate mentoring" [Title/Abstract] OR "postgraduate mentoring" [Title/Abstract] OR “effective mentors” [Title/Abstract] OR “trainers” [Title/Abstract] OR “trainer” [Title/Abstract] OR “instructors” [Title/Abstract] OR “instructor” [Title/Abstract] OR “educators” [Title/Abstract] OR “educator” [Title/Abstract]  ("mentoring"[MeSH Terms] OR "novice mentor*"[Title/Abstract] OR "undergraduate mentoring"[Title/Abstract] OR "postgraduate mentoring"[Title/Abstract] OR "effective mentors"[Title/Abstract] OR "trainers"[Title/Abstract] OR "trainer"[Title/Abstract] OR "instructors"[Title/Abstract] OR "instructor"[Title/Abstract] OR "educators"[Title/Abstract] OR "educator"[Title/Abstract]) AND ((2018/1/1:2022/12/31[pdat]) AND (english[Filter])) | 22,457  22,191 |
| #3 | "teacher training" [MeSH Terms] OR “train the trainer” [Title/Abstract] OR "mentoring training program" [Title/Abstract] OR “mentor training program” [Title/Abstract] OR "mentor training" [Title/Abstract] OR “mentoring training” [Title/Abstract] OR "training of mentors" [Title/Abstract] OR “training mentors” [Title/Abstract] OR “mentorship training” [Title/Abstract] OR “mentor development” [Title/Abstract] OR “mentor competence” [Title/Abstract] OR “mentors’ competence” [Title/Abstract] OR “mentoring education” [Title/Abstract] OR “mentorship training gap” [Title/Abstract] OR “mentoring gap” [Title/Abstract] OR “mentoring the mentor” [Title/Abstract] OR “preparing mentors” [Title/Abstract] OR “mentor preparation” [Title/Abstract] OR “mentor workshops” [Title/Abstract]  ("teacher training"[MeSH Terms] OR "train the trainer"[Title/Abstract] OR "mentor training program"[Title/Abstract] OR "mentor training"[Title/Abstract] OR "mentoring training"[Title/Abstract] OR "training of mentors"[Title/Abstract:~0] OR "training mentors"[Title/Abstract] OR "mentorship training"[Title/Abstract] OR "mentor development"[Title/Abstract] OR "mentor competence"[Title/Abstract] OR "mentors competence"[Title/Abstract] OR "mentoring education"[Title/Abstract] OR "mentoring gap"[Title/Abstract:~0] OR "mentoring the mentor"[Title/Abstract] OR "preparing mentors"[Title/Abstract] OR "mentor preparation"[Title/Abstract] OR "mentor workshop*"[Title/Abstract]) AND ((2018/1/1:2022/12/31[pdat]) AND (english[Filter])) | 830  822 |
| #4 | #1 AND #2 AND #3 | 117  125 |

(("students, medical"[MeSH Terms] OR "medical student"[Title/Abstract] OR "medical students"[Title/Abstract] OR "medical undergraduate students"[Title/Abstract] OR "medical postgraduate"[Title/Abstract] OR "medical postgraduates"[Title/Abstract] OR "physicians"[MeSH Terms] OR "physician"[Title/Abstract] OR "physicians"[Title/Abstract] OR "doctor"[Title/Abstract] OR "doctors"[Title/Abstract] OR "clinician"[Title/Abstract] OR "clinicians"[Title/Abstract] OR "resident"[Title/Abstract] OR "residents"[Title/Abstract] OR "medical practitioner"[Title/Abstract] OR "medical practitioners"[Title/Abstract] OR "consultants"[Title/Abstract] OR "consultant"[Title/Abstract] OR "attendings"[Title/Abstract] OR "attending"[Title/Abstract])) AND (("mentoring"[MeSH Terms] OR "novice mentor*"[Title/Abstract] OR "undergraduate mentoring"[Title/Abstract] OR "postgraduate mentoring"[Title/Abstract] OR "effective mentors"[Title/Abstract] OR "trainers"[Title/Abstract] OR "trainer"[Title/Abstract] OR "instructors"[Title/Abstract] OR "instructor"[Title/Abstract] OR "educators"[Title/Abstract] OR "educator"[Title/Abstract])) AND (("teacher training"[MeSH Terms] OR "train the trainer"[Title/Abstract] OR "mentor training program"[Title/Abstract] OR "mentor training"[Title/Abstract] OR "mentoring training"[Title/Abstract] OR "training of mentors"[Title/Abstract:~0] OR "training mentors"[Title/Abstract] OR "mentorship training"[Title/Abstract] OR "mentor development"[Title/Abstract] OR "mentor competence"[Title/Abstract] OR "mentors competence"[Title/Abstract] OR "mentoring education"[Title/Abstract] OR "mentoring gap"[Title/Abstract:~0] OR "mentoring the mentor"[Title/Abstract] OR "preparing mentors"[Title/Abstract] OR "mentor preparation"[Title/Abstract] OR "mentor workshop*"[Title/Abstract]))

2023 - 24 results (as of 12/3/24)

**Scopus (last searched on 14/1/22)**

*Publication date: 1/1/18-31/12/22

| #1 | TITLE-ABS-KEY((“medical students” OR “medical student” OR “medical students” OR “medical undergraduate students” OR “medical undergraduates students” OR “medical postgraduates” OR “medical postgraduates” OR “physicians” OR “physician” OR “physicians” OR “doctor” OR “doctors” OR “clinicians” OR “resident” OR “residents” OR “medical practitioner” OR “medical practitioners” OR “consultants” OR “consultants” OR “consultant” OR “attendings” OR “attending”) AND ("mentoring" OR "novice mentor" OR "undergraduate mentoring" OR "postgraduate mentoring" OR “effective mentors” OR “trainers” OR “trainer” OR “instructors” OR “instructor” OR “educators” OR “educator”) AND ("training of trainers" OR “train the trainer” OR "mentoring training program" OR “mentor training program” OR "mentor training" OR “mentoring training” OR "training of mentors" OR “training mentors” OR “mentorship training” OR “mentor development” OR “mentor competence” OR “mentors’ competence” OR “mentoring education” OR “mentorship training gap” OR “mentoring gap” OR “mentoring the mentor” OR “preparing mentors” OR “mentor preparation” OR “mentor workshops”)) PUBYEAR > 2017 AND PUBYEAR < 2023 LANGUAGE (english)  TITLE-ABS-KEY((“medical students” OR “medical student” OR “medical students” OR “medical undergraduate students” OR “medical postgraduate” OR “medical postgraduates” OR “physicians” OR “physician” OR “doctor” OR “doctors” OR “clinicians” OR “resident” OR “residents” OR “medical practitioner” OR “medical practitioners” OR “consultants” OR “consultant” OR “attendings” OR “attending”) AND ("mentoring" OR "novice mentor" OR "undergraduate mentoring" OR "postgraduate mentoring" OR “effective mentors” OR “trainers” OR “trainer” OR “instructors” OR “instructor” OR “educators” OR “educator”) AND ("training of trainers" OR “train the trainer” OR "mentoring training program" OR “mentor training program” OR "mentor training" OR “mentoring training” OR "training of mentors" OR “training mentors” OR “mentorship training” OR “mentor development” OR “mentor competence” OR “mentors’ competence” OR “mentoring education” OR “mentorship training gap” OR “mentoring gap” OR “mentoring the mentor” OR “preparing mentors” OR “mentor preparation” OR “mentor workshops”)) PUBYEAR > 2017 AND PUBYEAR < 2023 LANGUAGE (english) | 174 |
| --- | --- | --- |

TITLE-ABS-KEY ( ( "medical students" OR "medical student" OR "medical students" OR "medical undergraduate students" OR "medical postgraduate" OR "medical postgraduates" OR "physicians" OR "physician" OR "doctor" OR "doctors" OR "clinicians" OR "resident" OR "residents" OR "medical practitioner" OR "medical practitioners" OR "consultants" OR "consultant" OR "attendings" OR "attending" ) AND ( "mentoring" OR "novice mentor" OR "undergraduate mentoring" OR "postgraduate mentoring" OR "effective mentors" OR "trainers" OR "trainer" OR "instructors" OR "instructor" OR "educators" OR "educator" ) AND ( "training of trainers" OR "train the trainer" OR "mentoring training program" OR "mentor training program" OR "mentor training" OR "mentoring training" OR "training of mentors" OR "training mentors" OR "mentorship training" OR "mentor development" OR "mentor competence" OR "mentors&apos; competence" OR "mentoring education" OR "mentorship training gap" OR "mentoring gap" OR "mentoring the mentor" OR "preparing mentors" OR "mentor preparation" OR "mentor workshops" ) ) PUBYEAR = 2023 LANGUAGE ( english ) AND ( LIMIT-TO ( PUBYEAR , 2023 ) )

2023/4 - 32 articles (as of 12/3/24)

**Embase (last searched on 18/3/22)**

*Publication date: 1/1/18-31/12/22

| #1 | ‘medical student’/exp OR ‘medical student’:ti,ab OR ‘medical students’:ti,ab OR ‘medical undergraduate students’:ti,ab OR ‘medical undergraduates students’:ti,ab OR ‘medical postgraduates’:ti,ab OR ‘medical postgraduates’:ti,ab OR ‘physician’/exp OR ‘physician’:ti,ab OR ‘physicians’:ti,ab OR ‘doctor’:ti,ab OR ‘doctors’:ti,ab OR ‘clinicians’:ti,ab OR ‘resident’:ti,ab OR ‘residents’:ti,ab OR ‘medical practitioner’:ti,ab OR ‘medical practitioners’:ti,ab OR ‘consultants’:ti,ab OR ‘consultants’:ti,ab OR ‘consultant’:ti,ab OR ‘attendings’:ti,ab OR ‘attending’:ti,ab  ‘medical student’/exp OR ‘medical student’:ti,ab OR ‘medical students’:ti,ab OR ‘medical undergraduate students’:ti,ab OR ‘medical postgraduates’:ti,ab OR ‘physician’/exp OR ‘physician’:ti,ab OR ‘physicians’:ti,ab OR ‘doctor’:ti,ab OR ‘doctors’:ti,ab OR ‘clinicians’:ti,ab OR ‘clinician’:ti,ab OR ‘resident’:ti,ab OR ‘residents’:ti,ab OR ‘medical practitioner’:ti,ab OR ‘medical practitioners’:ti,ab OR ‘consultants’:ti,ab OR ‘consultant’:ti,ab OR ‘attendings’:ti,ab OR ‘attending’:ti,ab | 2,262,010 |
| --- | --- | --- |
| #2 | ‘mentoring’/exp OR ‘novice mentor’:ti,ab OR ‘undergraduate mentoring’:ti,ab OR ‘postgraduate mentoring’:ti,ab OR ‘effective mentors’:ti,ab OR ‘trainers’:ti,ab OR ‘trainer’:ti,ab OR ‘instructors’:ti,ab OR ‘instructor’:ti,ab OR ‘educators’:ti,ab OR ‘educator’:ti,ab | 78,096 |
| #3 | ‘teacher training’/exp OR ‘train the trainer’:ti,ab OR ‘mentoring training program’:ti,ab OR ‘mentor training program’:ti,ab OR ‘mentor training’:ti,ab OR ‘mentoring training’:ti,ab OR ‘training of mentors’:ti,ab OR ‘training mentors’:ti,ab OR ‘mentorship training’:ti,ab OR ‘mentor development’:ti,ab OR ‘mentor competence’:ti,ab OR ‘mentors competence’:ti,ab OR ‘mentoring education’:ti,ab OR ‘mentorship training gap’:ti,ab OR ‘mentoring gap’:ti,ab OR ‘mentoring the mentor’:ti,ab OR ‘preparing mentors’:ti,ab OR ‘mentor preparation’:ti,ab OR ‘mentor workshops’:ti,ab | 2513 |
| #4 | #1 AND #2 AND #3 AND [2018-2022]/py AND [embase]/lim NOT ([embase]/lim AND [medline]/lim) AND [english]/lim | 137 |

**('medical student'/exp OR 'medical student':ti,ab OR 'medical students':ti,ab OR 'medical undergraduate students':ti,ab OR 'medical postgraduates':ti,ab OR 'physician'/exp OR 'physician':ti,ab OR 'physicians':ti,ab OR 'doctor':ti,ab OR 'doctors':ti,ab OR 'clinicians':ti,ab OR 'clinician':ti,ab OR 'resident':ti,ab OR 'residents':ti,ab OR 'medical practitioner':ti,ab OR 'medical practitioners':ti,ab OR 'consultants':ti,ab OR 'consultant':ti,ab OR 'attendings':ti,ab OR 'attending':ti,ab) AND ('mentoring'/exp OR 'novice mentor':ti,ab OR 'undergraduate mentoring':ti,ab OR 'postgraduate mentoring':ti,ab OR 'effective mentors':ti,ab OR 'trainers':ti,ab OR 'trainer':ti,ab OR 'instructors':ti,ab OR 'instructor':ti,ab OR 'educators':ti,ab OR 'educator':ti,ab) AND ('teacher training'/exp OR 'train the trainer':ti,ab OR 'mentoring training program':ti,ab OR 'mentor training program':ti,ab OR 'mentor training':ti,ab OR 'mentoring training':ti,ab OR 'training of mentors':ti,ab OR 'training mentors':ti,ab OR 'mentorship training':ti,ab OR 'mentor development':ti,ab OR 'mentor competence':ti,ab OR 'mentors competence':ti,ab OR 'mentoring education':ti,ab OR 'mentorship training gap':ti,ab OR 'mentoring gap':ti,ab OR 'mentoring the mentor':ti,ab OR 'preparing mentors':ti,ab OR 'mentor preparation':ti,ab OR 'mentor workshops':ti,ab) AND [2023-2024]/py AND [embase]/lim NOT ([embase]/lim AND [medline]/lim) AND [english]/lim**

2023 results - 34 (as of 12/3/24)

**PsycINFO (last searched on 26/3/23)**

*Publication date: 1/1/18-31/12/22

| S/N | Search Terms | # of results |
| --- | --- | --- |
| 1 | exp Medical Students/ | 14734 |
| 2 | exp Physicians/ | 48510 |
| 3 | 1 or 2 | 61838 |
| 4 | (medical students or medical student or medical students or medical undergraduate students or medical undergraduates students or medical postgraduates or medical postgraduates or physicians or physician or physicians or doctor or doctors or clinician or clinicians or resident or residents or medical practitioner or medical practitioners or consultants or consultant or attendings or attending).ti,ab | 297617 |
| 5 | exp Mentor/ | 8054 |
| 6 | (mentor or mentoring or novice mentor or undergraduate mentoring or postgraduate mentoring or effective mentors or trainers or trainer or instructors or instructor or educators or educator).ti,ab | 101431 |
| 7 | exp Training | 89141 |
| 8 | Exp teacher education/ | 19460 |
| 9 | (training or training of trainers or train the trainer or mentoring training program or mentor training program or mentor training or mentoring training or training of mentors or training mentors or mentorship training or mentor development or mentor competence or mentors competence or mentoring education or mentorship training gap or mentoring gap or mentoring the mentor or preparing mentors or mentor preparation or mentor workshops).ti,ab | 291033 |
| 10 | 3 or 4 | 318054 |
| 11 | 5 or 6 | 102562 |
| 12 | 7 or 8 or 9 | 325796 |
| 13 | 10 and 11 and 12 | 2979 |
| 14 | limit 13 to (english language and yr=“2018 = 2022”) | 602 |

(exp Medical Students/ or exp Physicians/ or (medical students or medical studen or medical students or medical undergraduate students or medical undergraduates students or medical postgraduates or medical postgraduates or physicians or physician or physicians or doctor or doctors or clinicians or resident or residents or medical practitioner or medical practitioners or consultants or consultants or consultant or attendings or attending).ti,ab) **and (exp Mentor/ or (mentor or mentoring or novice mentor or undergraduate mentoring or postgraduate mentoring or effective mentors or trainers or trainer or instructors or instructor or educators or educator).ti,ab)** and (exp Training/ or (training or training of trainers or train the trainer or mentoring training program or mentor training program or mentor training or mentoring training or training of mentors or training mentors or mentorship training or mentor development or mentor competence or mentors competence or mentoring education or mentorship training gap or mentoring gap or mentoring the mentor or preparing mentors or mentor preparation or mentor workshops).ti,ab)

2023/4 articles - 111 (as of 12/3/24)

**CINAHL (last searched on 30/1/23)**

*Publication date: 1/1/18-31/12/22

| #1 | ((MH “Students, Medical+”) OR (MH “Physicians+”) OR (TI “medical student” OR AB “medical student”) OR (TI “medical undergraduate students” OR AB “medical undergraduate students”) OR (TI “medical undergraduates students” OR AB “medical undergraduates students”) OR (TI “medical postgraduates” OR AB “medical postgraduates”) OR (TI “physicians” OR AB “physicians”) OR (TI “physician” OR AB “physician”) OR (TI “physicians” OR AB “physicians”) OR (TI “doctor” OR AB “doctor”) OR (TI “doctors” OR AB “doctors”) OR (TI “clinicians” OR AB “clinicians”) OR (TI “resident” OR AB “resident”) OR (TI “residents” OR AB “residents”) OR (TI “medical practitioner” OR AB “medical practitioner”) OR (TI “medical practitioners” OR AB “medical practitioners”) OR (TI “” OR AB “”) OR (TI “” OR AB “”) OR (TI “consultants” OR AB “consultants”) OR (TI “consultant” OR AB “consultant”) OR (TI “attendings” OR AB “attendings”) OR (TI “attending” OR AB “attending”)) | 158, 270 |
| --- | --- | --- |
| #2 | ((MH “Mentorship+”) OR (TI “novice mentor” OR AB “novice mentor”) OR (TI “undergraduate mentoring” OR AB “undergraduate mentoring”) OR (TI “postgraduate mentoring” OR AB “postgraduate mentoring”) OR (TI “effective mentors” OR AB “effective mentors”) OR (TI “trainers” OR AB “trainers”) OR (TI “trainer” OR AB “trainer”) OR (TI “instructors” OR AB “instructors”) OR (TI “instructor” OR AB “instructor”) OR (TI “educators” OR AB “educators”) OR (TI “educator” OR AB “educator”)) | 17, 914 |
| #3 | ((TI “train the trainer” OR AB “train the trainer”) OR (TI “mentoring training program” OR AB “mentoring training program”) OR (TI “mentor training program” OR AB “mentor training program”) OR (TI “mentor training” OR AB “mentor training”) OR (TI “mentoring training” OR AB “mentoring training”) OR (TI “training of mentors” OR AB “training of mentors”) OR (TI “training mentors” OR AB “training mentors”) OR (TI “mentorship training” OR AB “mentorship training”) OR (TI “mentor development” OR AB “mentor development”) OR (TI “mentor competence” OR AB “mentor competence”) OR (TI “mentors’ competence” OR AB “mentors’ competence”) OR (TI “mentoring education” OR AB “mentoring education”) OR (TI “mentorship training gap” OR AB “mentorship training gap”) OR (TI “mentoring gap” OR AB “mentoring gap”) OR (TI “mentoring the mentor” OR AB “mentoring the mentor”) OR (TI “preparing mentors” OR AB “preparing mentors”) OR (TI “mentor preparation” OR AB “mentor preparation”) OR (TI “mentor workshops” OR AB “mentor workshops”) | 296 |
| #4 | #1 AND #2 AND #3 | 58 |

**(((MH “Students, Medical+”) OR (MH “Physicians+”) OR (TI “medical student” OR AB “medical student”) OR (TI “medical undergraduate students” OR AB “medical undergraduate students”) OR (TI “medical undergraduates students” OR AB “medical undergraduates students”) OR (TI “medical postgraduates” OR AB “medical postgraduates”) OR (TI “physicians” OR AB “physicians”) OR (TI “physician” OR AB “physician”) OR (TI “physicians” OR AB “physicians”) OR (TI “doctor” OR AB “doctor”) OR (TI “doctors” OR AB “doctors”) OR (TI “clinicians” OR AB “clinicians”) OR (TI “resident” OR AB “resident”) OR (TI “residents” OR AB “residents”) OR (TI “medical practitioner” OR AB “medical practitioner”) OR (TI “medical practitioners” OR AB “medical practitioners”) OR (TI “” OR AB “”) OR (TI “” OR AB “”) OR (TI “consultants” OR AB “consultants”) OR (TI “consultant” OR AB “consultant”) OR (TI “attendings” OR AB “attendings”) OR (TI “attending” OR AB “attending”))) AND (((MH “Mentorship+”) OR (TI “novice mentor” OR AB “novice mentor”) OR (TI “undergraduate mentoring” OR AB “undergraduate mentoring”) OR (TI “postgraduate mentoring” OR AB “postgraduate mentoring”) OR (TI “effective mentors” OR AB “effective mentors”) OR (TI “trainers” OR AB “trainers”) OR (TI “trainer” OR AB “trainer”) OR (TI “instructors” OR AB “instructors”) OR (TI “instructor” OR AB “instructor”) OR (TI “educators” OR AB “educators”) OR (TI “educator” OR AB “educator”))) AND (((TI “train the trainer” OR AB “train the trainer”) OR (TI “mentoring training program” OR AB “mentoring training program”) OR (TI “mentor training program” OR AB “mentor training program”) OR (TI “mentor training” OR AB “mentor training”) OR (TI “mentoring training” OR AB “mentoring training”) OR (TI “training of mentors” OR AB “training of mentors”) OR (TI “training mentors” OR AB “training mentors”) OR (TI “mentorship training” OR AB “mentorship training”) OR (TI “mentor development” OR AB “mentor development”) OR (TI “mentor competence” OR AB “mentor competence”) OR (TI “mentors’ competence” OR AB “mentors’ competence”) OR (TI “mentoring education” OR AB “mentoring education”) OR (TI “mentorship training gap” OR AB “mentorship training gap”) OR (TI “mentoring gap” OR AB “mentoring gap”) OR (TI “mentoring the mentor” OR AB “mentoring the mentor”) OR (TI “preparing mentors” OR AB “preparing mentors”) OR (TI “mentor preparation” OR AB “mentor preparation”) OR (TI “mentor workshops” OR AB “mentor workshops”))**

**2023/24 articles - 8 (as of 12/3/24)**
