# Supplementary material for: A systematic scoping review of mentor training in medical education between 2000 and 2024
Source: BMC Med Educ. 2025 Jul 24;25:1110. doi: 10.1186/s12909-025-07353-x (PMC12291282; doi:10.1186/s12909-025-07353-x)
Supplement: Supplementary file 3 — Additional file 3. Tabulated Summary of Included Articles. [file 12909_2025_7353_MOESM3_ESM.docx]

**Additional File 3. Tabulated Summaries of Included Articles**

| **S/N** | **Author (Year)** | **Article Title** | **Type of study** | **Study details**  **(N = number of mentors)** | **Study aims and methodology** | **Intervention for mentors** | **Findings** | **Insights/ Conclusions** |
| --- | --- | --- | --- | --- | --- | --- | --- | --- |
| 1 | Abedin et al. (2012) | Deriving Competencies for Mentors of Clinical and Translational Scholars | Qualitative | Review of role of mentorship in clinical and translational science investigators | Although the importance of research mentorship has been well established, the role of mentors of junior clinical and translational science investigators is not clearly defined. The authors attempt to derive a list of actionable competencies for mentors from a series of complementary methods. They examined focus groups, the literature, competencies derived for clinical and translational scholars, mentor  training curricula, mentor evaluation forms and finally conducted an expert panel process in order to compose this list. | NIL | These efforts resulted in a set of competencies that include generic competencies expected of all mentors, competencies specific to scientists, and competencies that are clinical and translational research specific. They are divided into six thematic areas: (1) Communication and managing the relationship, (2) Psychosocial support, (3) Career and professional development, (4) Professional enculturation and scientific integrity, (5) Research development, and (6) Clinical and translational investigator development. For each thematic area, the authors have listed associated competencies, 19 in total. For each competency, examples that are actionable and measurable have been listed. | Although a comprehensive approach was used to derive this list of competencies, further work will be required to parse out how to apply and adapt them, as well future research directions and evaluation processes. |
| 2 | Abedin et al. (2013) | Mentor Training within Academic Health Centers with Clinical and  Translational Science Awards | Qualitative | Review of mentoring practices at academic health centers with Clinical and Translational Science Awards | Multiple studies highlight the benefits of effective mentoring in academic medicine. Thus, the authors sought to quantify and characterize the mentoring practices at academic health centers (AHCs) with Clinical and Translational Science Awards (CTSA).  Findings pertaining specifically to mentor training at the level of the KL2 mentored award program, and at the broader institutional level have been reported. We found only four AHCs did not provide any form of training. | NIL | One-time orientation was most prevalent at the KL2 level, whereas formal  face-to-face training was most prevalent at the institutional level. Despite differences in format usage, there was general consensus at both the KL2 and institutional level about the topics of focus of face-to-face training sessions. Lower-resource training formats utilized at the KL2 level may reveal a preference for preselection of qualified mentors, while institutional selection of resource-heavy formats may  be an attempt to raise the mentoring qualifications of the academic community as a whole. | The present work fits into the expanding landscape of academic mentoring literature and sets the framework for future longitudinal, outcome studies focused on identifying the  most efficient strategies to develop effective mentors. |
| 3 | Adame et al. (2019) | Training the Anesthesiologist Trainer: Enhancing the Quality of Feedback during Human Patient Simulations | Randomised controlled trial | Control group, or mentors received feedback training.  N=50 | An essential piece of anesthesiologist training is attending resident feedback sessions. Yet, few attending  anesthesiologists have formal teaching education and little time to acquire it.  In this field experiment, attending physicians were randomly assigned to a control group or to receive 30 minutes of  feedback training inspired by Implicit Person Theory (IPT). As such, IPT training encouraged physicians to  praise process-oriented learning while discouraging performance-oriented mindsets. Attending physicians  then observed residents participate in a human patient simulation (HPS) activity and provided residents with feedback. | 30 minutes of training | Content and statistical analyses revealed trained attending physicians praised learning goals and challenged performance goals more often than untrained physicians during feedback sessions. | Thus, the training provides a rapid method of enhancing the quality of attending physicians’  training communication. |
| 4 | Aliyu et al. (2022) | Building Research Capacity in HIV and Noncommunicable Diseases in Africa: A Mentorship and Leadership Workshop Report | Feasibility study | Early-stage physician scientists in Nigeria trained to mentor regarding HIV.  N=33 | Few structured mentoring programs target early-stage investigators in Africa, creating a gap in mentorship skills where HIV burden is greatest. This article describes findings from a Nigeria-based workshop for early-career physician scientists to build mentoring and leadership capacity in HIV and noncommunicable disease research.  Baseline surveys captured participant demographics, confidence in implementing mentoring competencies, and perceived importance of workshop training domains. The workshop included didactic presentations, small group activities, and interactive discussions. Daily surveys evaluated sessions, and postworkshop surveys solicited overall course impressions. | 4-day training session | Of the 33 participants, most were male (n = 21, 63.6%) and from medicine, laboratory sciences, and surgical specialties. “Building mentees’ confidence” and “setting mentees’ research goals” were ranked as areas where participants most believed they needed training. Sessions were rated favorably across five areas. Greatest improvements in mean scores were for confidence in identifying personal temperament styles, describing mentoring and leadership theories/frameworks, and developing mentoring plans. Additional identified workshop strengths were content relevance, leadership case series, interactive nature, and collegial atmosphere. All respondents indicated learning something new/useful/helpful in each session. At 6-month postworkshop, most respondents (25 of 26, 96%) had replicated or plan to replicate parts of the workshop in their departments/institutions. | Effective mentoring training initiatives targeting future academic leaders have the potential to create skilled academicians who can impart mentoring skills and competencies to their mentees. |
| 5 | Anderson and Vanderbilt (2018) | Bridging the gap between physician and medical student education: using the Train the Trainer model to improve cultural competence training in the clerkship years of medical school | Scoping Review | Proposition of cultural competence training delivered through “Train-the-Trainer” model | Current cultural competence (CC) training seems to be inadequate as research has demonstrated gaps between CC training and clinical behaviors of students. One aspect that is potentially contributing to this gap is the lack of physician education of CC. To change this, the authors propose the implementation of a Train the Trainer model in which the preclinical professor in charge of CC education trains Clerkship and Residency Directors who then can train and supervise the physicians and residents in their departments on CC to better implement it into the formal and informal curriculum of clerkships. | NIL | Many TtT modeled seminars found those who participated not only had significant increases in knowledge demonstrated by the change in their pre- and postevaluations, but were also successful in teaching the information to others. Several studies have shown that when comparing TtT to self-study or traditional lecture-style CME training, TtT resulted in greater improvement in performance and adherence to the skill being learned. There is also reported increase in confidence in developing and teaching a CC curriculum. | This is an efficient, cost-effective method of imparting cultural competence to physicians during their early years. |
| 6 | Anderson et al. (2012) | Evaluating and Giving Feedback to Mentors: New Evidence-Based Approaches | Scoping Review | Creation of an approach for mentor evaluation | A comprehensive mentoring program includes a variety of components. One of the most important is the ongoing assessment of and feedback to mentors. Scholars need strong active mentors who have the expertise, disposition, motivation, skills, and the ability to accept feedback and to adjust their mentoring style. Assessing the effectiveness of a given mentor is no easy task. Variability in learning needs and academic goals among scholars makes it difficult to develop a single evaluation instrument or a standardized procedure for evaluating mentors. Scholars, mentors, and program leaders are often reluctant to conduct formal evaluations, as there are no commonly accepted measures.The process of giving feedback is often difficult and there is limited empirical data on efficacy.  This article presents a new and innovative six-component approach to mentor evaluation that includes the assessment of mentee training and empowerment, peer learning and mentor training, scholar advocacy, mentee–mentor expectations, mentor self-reflection, and mentee  evaluation of their mentor. | NIL | Mentor evaluation should involve assessment of mentee empowerment and training, mentee evaluation of the mentor, peer learning and mentor training, together with expectations, self-reflection of the mentor. | The primary goals of mentor and programmatic evaluation are to (1) increase learning opportunities for scholars, (2) assist mentors to become stronger mentors, and (3) guide scholar training activities. This article presents a more comprehensive approach to evaluation that tries to connect a number of important elements of comprehensive mentoring program. As in clinical medicine, where ongoing evaluation of clinical performance is becoming the norm, training young investigators deserves the same level of effort and use of new evaluation models. |
| 7 | Avery and Cowburn (2023) | Training the trainers: improving the quality of education delivered to paramedics through a simulation-debrief model | Qualitative | Usage of senior doctors to design train-the-trainer course.  N=48 | The South Western Ambulance  Service NHS Foundation Trust employs senior doctors in their learning and development (L&D) team to support the development of ‘train the trainer’ courses for L&D officers (LDOs). This short  report of a quality improvement initiative describes the implementation and evaluation of a simulation-debrief model of paramedic education.  A quality improvement design was adopted. The train the trainer scenarios for simulation-debrief were designed and written following the trust’s training needs analysis by the L&D team. The course ran for two days, and each scenario was facilitated by faculty experienced  in simulation (both doctors and paramedics). Low-fidelity mannequins and standard ambulance  training kit was used (including response bags, training monitor and defibrillator). Participants’ pre- and post-scenario self-reported confidence scores were recorded, and qualitative feedback  requested. Numerical data were analysed, and collated into graphs using Excel. Thematic analysis of comments was used to present qualitative themes. The SQUIRE 2.0 checklist for reporting  quality improvement initiatives was used to frame this short report. | 2-day course | Forty-eight LDOs attended across three courses. All participants reported improved confidence scores in the clinical topic covered after each simulation-debrief scenario, with a minority reporting equivocal scores. Formal qualitative feedback from participants indicated  an overwhelmingly positive response to the introduction of simulation-debrief as an education  method, and a move away from summative, assessment-based training. The positive value of a  multidisciplinary faculty was also reported. | The simulation-debrief model of paramedic education represents a move away from the use of didactic teaching and ‘tick box’-style assessments in previous train the trainer courses. The introduction of simulation-debrief teaching methodology has had a positive impact on paramedics’ confidence in the selected clinical topics, and is seen by LDOs as an effective and  valuable education method. |
| 8 | Blanchard et al. (2015) | Cultivating Medical Education Research Mentorship as a Pathway Towards High Quality Medical Education Research | Qualitative | Review of research mentor training and mentorship in undergraduate and graduate medical education | This paper explores the integration of doctoral-level educators, structured inter-departmental efforts, and external mentorship to provide opportunities for faculty to gain traction in their medical education research efforts. An investment in medical education research mentors will ensure rigorous research for high quality innovation in medical education and patient care. | NIL | Investment into medical education research mentors, via means such as mentor training improves research quality, improving medical education and patient care.  One opportunity for improved mentorship is to employ a PhD-educator. Second, inter-departmental collaboration should be encouraged to create a recognized forum for mentorship and collaboration in Medical Education Research (MER). More locally, program directors and core faculty can encourage residents and junior faculty to seek external mentorship for research when they are unable to provide it themselves. Alternatively, a curriculum in MER can be implemented. | Creating adequate career paths for clinician educators to become education research mentors is necessary in order to encourage progress in medical education. Developing highly trained researchers ensures mentorship for continued high quality MER, necessary for robust innovations in medical education and patient care. |
| 9 | Blatt et al. (2018) | Preparing interprofessional faculty to be humanistic mentors for medical students: The GW-gold mentor development program | Feasibility study | GW-Gold Humanistic Mentor Development Programme to trains faculty mentoring teams in humanistic communities of practice.  N=44 | The GW-Gold Humanistic Mentor Development Program addresses the challenge faced by medical schools to  educate faculty to prepare students for humanistic practice. Grounded in Branch’s Teaching Professional and Humanistic Values  model, the program prepares interprofessional faculty mentoring teams in humanistic communities of practice. The teams consist  of physician-psychosocial professional pairs, each mentoring a small student group in their professional development course.  Through GW-Gold workshops, faculty mentors develop interprofessional humanistic communities of practice, preparing them to  lead second such communities with their students. This article describes the program and its evaluation.  To assess outcomes and better understand the mentor experience, we used a mixed-method validating triangulation  design consisting of simultaneous collection of quantitative (mentor and student surveys) and qualitative (open-ended survey  questions and focus group) data. Data were analyzed in parallel and merged at the point of interpretation, allowing for triangulation  and validation of outcomes. | Monthly workshops | Mentors rated the program highly, gained confidence in their humanistic skills, and received high scores from students.  Three themes emerged that validated program design, confirmed outcomes, and expanded on the mentor experience: (1)  Interprofessional faculty communities developed through observation, collaboration, reflection, and dialogue; (2) Humanistic  mentors created safe environments for student engagement; and (3) Engaging in interprofessional humanistic communities of  practice expanded mentors’ personal and professional identities. | Outcomes support the value of the GW-Gold program’s distinctive features in preparing faculty to sustain humanism  in medical education: an interprofessional approach and small communities of practice built on humanistic values. |
| 10 | Bunin and Servey. (2019) | Meeting the needs of clinician-educators: An innovative faculty development community of practice | Feasibility study | Train-the-trainer programme to develop faculty, nurturing community of practice.  N=16,729 | Requirements for faculty development for clinician-educators continue to increase. The number of faculty with experience delivering faculty development, however, remains limited. To overcome their deficit of faculty developers, the authors created a train-the-trainer programme. They recognised, however, that their plan would not meet the ultimate goal for our programme: Creating faculty developers to meet the faculty development needs of a large, dispersed system. The article reports on the creation and nurturing of faculty development community of practice (CoP), which they believe could be a solution for many academic systems struggling to engage busy clinicians, mature the teaching abilities of clinician educators, and meet the needs of their accrediting institutions.  The  faculty development CoP was developed with a mission of promoting educational expertise and excellence and ensuring continuous growth of the members of our COP and broader faculty. | 21 standardised workshop formats, 1188 workshops delivered to 16,729 attendees | A programme evaluation was performed consisting of two elements: The impact on the organisation (workshop and learner related metrics) and the impact on the CoP members (survey). Notable outcomes were observed: Delivery of high quality workshops to faculty, attainment of leadership positions, and increases in motivation, networking, skills, confidence, and opportunities available to members.  Personal and professional identities developed, creating safe environments for engagements and faculty communities were developed. | Innovations to create and sustain structured faculty development  programmes for clinician-educators are needed. The development and nurturing of a community of practice  (CoP) of faculty developers resulted in benefits both for the organisation and CoP  members and may be a solution for large academic systems struggling to meet their  faculty development demands. |
| 11 | Butki et al. (2020) | A Novel ‘Train the Trainer’ Emergency Medicine Resident Point-of-Care Ultrasound Course: A Feasibility Study | Feasibility study | Novices, to become mentors, underwent training in point-of care ultrasound.  N=42 | The specific aim of this study was to explore the feasibility of using volunteer EM physicians who were novices with ultrasound techniques as instructors for a POCUS course. Additionally, the authors evaluated the effectiveness and consistency of a POCUS course delivered over multiple sites to enhance EM residents’ ultrasound knowledge and skill acquisition.  For the initial session, the lead instructor conducted a focused two-hour course with the novice instructors. A subsequent four-hour session was then repeated for EM residents whereby the aforementioned novice instructors provided the hands-on instruction. The residents were given 10-item pre- and 20-item post-course knowledge tests to gauge the effectiveness of the instruction model. After the course, a satisfaction survey was administered to the resident participants and a qualitative open-ended survey to the volunteer EM physicians who served as instructors. | 2-hour training | Forty-two EM residents from 11 different residency programs attended at one of the three courses that were offered. After adjustments for size differences in the pre- and post-training tests, 35 (87.5%) of total sample resident learners’ scores proportionately increased from pre- to post-test scores, with five (11.9%) other residents maintaining their pre-course score levels and only two (4.8%) residents experienced a post-score decline. In addition, resident participants responded favorably to a post-course summary evaluation with an average response of 4.8 (0-5 Likert scale) demonstrating overall satisfaction with the course. In the separate qualitative survey given to instructors, comments consistently conveyed a perceived benefit for the volunteer EM physicians.  Generally, skills improved, allowing novices to become effective educators | The evaluation of this novel model supports the feasibility of the ‘train the trainer’ program. It provides a proof of principle that train the trainer model can be implemented for POCUS training courses. Despite the small sample size, the results show an increase in the pre- to post-test scores among most participating residents. This model provides an additional option for EM residency program educators to consider when developing their POCUS training courses across multiple GME settings. |
| 12 | Buxton et al. (2023) | Peer-to-Peer Trauma-Informed Training for Surgical Residents Facilitated by Psychiatry Residents | Feasibility study | Mentors trained in trauma-informed care, then developed training curriculum for others.  N=10 | This article describes the implementation of trauma-informed care (TIC) didactic training, using a novel, interdisciplinary peer-to-peer teaching model to improve confidence surrounding trauma-informed practices in a surgical residency program.  Eight psychiatry residents and two medical students with a background in psychological trauma and TIC and an interest in medical education were recruited to participate in three 2-hour “train the trainer” sessions led by a national expert in TIC. Eight psychiatry residents and two medical students subsequently developed and delivered the initial TIC training to 29 surgical interns. Training included the neurobiology of psychological trauma, principles of trauma-informed care, and developing trauma-informed curricula. | Three 2-hour sessions | Surgical interns reported significantly improved understanding of the physiology of trauma, knowledge of TIC approaches, and confidence and comfort with TIC and practices. Among surgical interns, understanding of the physiology of the fear response increased from 3.36 to 3.85 (p = 0.03). Knowledge of the neurobiology of trauma improved between pre- and post-training surveys (2.71 to 3.64, p = 0.006). Surgery interns also expressed an improved understanding of the connection between fear, trauma, and aggression (3.08 to 4.23, p = 0.002) from pre- to post-training surveys. Post-training knowledge of trauma-informed approaches increased from 2.57 to 4.71 (p < 0.001) and confidence in delivering TIC on the wards increased from 2.79 to 4.64 (p < 0.001). | This TIC curriculum delivered via a peer-to-peer training model presents an effective way to improve comfort and confidence surrounding TIC practices and approaches in a surgical residency training program. |
| 13 | Chen et al. (2016) | A Multi-faceted Mentoring Program for Junior Faculty in Academic Pediatrics | Qualitative | Implementation of mentoring programme in an academic pediatric department  N=8 | The departure of physician-scientists from education and research into clinical practice is a growing challenge for the future of academic medicine. Junior faculty face competing demands for clinical productivity, teaching, research and work-life integration which can undermine confidence in the value of an academic career. Mentorship is important to foster career development and satisfaction in junior faculty.  The goals of this academic pediatrics department were to develop, implement, and evaluate a multi-faceted pediatric mentoring program to promote retention and satisfaction of junior faculty. Program elements included one-on-one mentor-mentee meetings, didactic workshops, grant review assistance, and facilitated peer-group mentoring. Program effectiveness was assessed using annual surveys of mentees, structured mentee exit interviews as well as retention data for assistant professors. | Initial training for mentors and division chiefs, followed by monthly meetings | Seventy-nine mentees participated in the program from 2007 through 2014. The response rate from seven annual surveys was 84%. Sixty-nine percent of mentees felt more prepared to advance their careers, 81% had a better understanding of the criteria for advancement, 84% were satisfied with the program, and 95% found mentors accessible. Mentees who exited the program reported they most valued the one-on-one mentoring and viewed the experience positively regardless of promotion. Retention of Assistant Professors improved after initiation of the program; 4 of 13 hired from 2002–2006 left the institution whereas 18 of 18 hired from 2007–2014 were retained. | This multi-faceted mentoring program appeared to bolster satisfaction and enhance retention of junior pediatric faculty. Mentees reported increased understanding of the criteria for promotion and viewed the program as a positive experience regardless of career path. Individual mentor-mentee meetings were needed at least twice yearly to establish the mentoring relationship. Identifying “next steps” at the end of individual meetings was helpful to hold both parties accountable for progress. Mentees most valued workshops fostering development of tangible skills (such as scientific writing) and those clarifying the criteria for promotion more transparent. Facilitated peer-group mentoring for mentees at the Instructor rank provided valuable peer support. |
| 14 | Collins et al. (2019) | Utilising the Delphi Process to Develop a Proficiency-based Progression Train-the-trainer Course for Robotic Surgery Training | Scoping review | Design of a train-the-trainer programme for robot-assisted surgery, following a systematic review | As the role of robot-assisted surgery continues to expand, development o  standardised and validated training programmes is becoming increasingly important.  The project was carried out in phases: a systematic review of the  current evidence was conducted, a face-to-face meeting was held in Philadelphia, and then an initial survey was created based on the current literature and expert opinion and sent to the committee. Thirty-two experts in training, including clinicians, academics,  and industry, contributed to the Delphi process. The Delphi process underwent three rounds of survey in total. Additions to the second- and third-round surveys were formulated based on the answers and comments from the previous rounds. Consensus  opinion was defined as 80% agreement. | NIL | There was 100% consensus that there was a need for a standardized TTT course in robotic surgery. Curriculum was formatted based on definitions and terminologies, qualifications to attend, course objectives, precourse considerations, requirement of e-learning, theory and course content, and measurement of outcomes and performance level verification.  The resulting formulated curriculum showed good internal consistency among experts, with a Cronbach alpha of 0.90. | Using the Delphi methodology, an international consensus was achieved among experts to develop and reach content validation for a standardised TTT curriculum for robotic surgery training. This defined content lays the foundation for developing a proficiency-based progression model for trainers in robotic surgery. |
| 15 | Connor et al. (2000) | Developing senior doctors as mentors: a form of continuing professional development. Report of an initiative to develop a network of senior doctors as mentors | Qualitative | Mentoring network in consultants and general practitioners  N=116 | Senior doctors report that mentoring skills are transferable to everyday medical practice and managing juniors. An analysis of views from consultants and general practitioners, who had trained together on a regional mentoring scheme, reveals significant potential for personal and professional  development in such networks.  The Northern and Yorkshire Region Doctors' Development and Mentoring Network was set up in 1994. Since then there have been six programmes with   116 senior doctors participating. In 1997 there was an evaluation of the first four programmes. Through focus groups and postal questionnaire, the authors evaluated these mentoring programmes and network. | Monthly workshops for 6 months | Results indicate that the programmes were highly valued by the participants, particularly with regard to: being part of a network of senior doctors; developing mentoring skills, and engaging in personal and professional development.   The most difficult part of the programme was setting   up mentoring networks for junior doctors, and reasons   included: personal factors, such as levels of confidence   in providing mentoring; cultural factors, such as juniors   not wishing to be seen to need help, and organizational   factors, such as lack of time allocated for mentoring. | The positive benefits from the scheme raise questions about  how to develop mentoring training for senior doctors.  Issues include: developing mentors; who needs men-  toring; mentoring and the organization; transferability  of mentoring skills, and widening the network. |
| 16 | Cramer et al. (2018) | Teach the Teacher: Training Otolaryngology Fellows to Become  Academic Educators | Feasibility study | Mentors trained in teaching skills for otolaryngology – head and neck surgery.  N=30 | Fellowship is the capstone of academic training and serves as preparation for an academic  career. Fellows are expected to educate medical students and residents during and long after fellowship. However, little time  is typically spent teaching fellows to become effective educators. The authors investigate a formal curriculum addressing teaching skills among fellows in otolaryngology–head and neck surgery (OHNS). They developed and implemented an educational program called Teach the Teacher to build skills as educators  for fellows in OHNS. They conducted a survey of fellows from 2014 to 2017 in OHNS who participated in the course. The survey evaluated demographics, teaching experiences, and teaching limitations structured as yes/no and Likert-style questions | Longitudinal programme | Thirty fellows were surveyed with a response rate was 80%. Fellowship was rated highly as an experience that  will make fellows a better academic educator (mean standard deviation: 4.5460.64). The most important components of teaching during fellowship were role modeling (4.6760.62), followed by teaching psychomotor skills in the operating room  (4.2960.89), diagnostic reasoning (4.2560.66), and evidence-based medicine (4.2560.83). The Teach the Teacher course specifically was rated as a helpful experience (4.0060.90). The primary limitations to developing teaching skills during fellowship  identified were lack of time, patient safety, and inexperience with hospital culture. | Fellowship is a key time to improve skills as academic educators. Fellows value formal efforts to teach academic  skills. |
| 17 | Crow et al. (2018) | A "Fundamentals" Train-the-Trainer Approach to Building Pediatric Critical Care Expertise in the Developing World | Feasibility study | Pediatric Fundamental Critical Care Support, an educational tool, taught to locals in Georgia who then conducted the course for locals.  N=6 | Pediatric Fundamental Critical Care Support (PFCCS) is an educational tool for training non-intensivists, nurses, and critical care practitioners in diverse health-care settings to deal with the acute deterioration of pediatric patients. The objective of this paper was to evaluate the PFCCS course as a tool for developing a uniform, reproducible, and sustainable model for educating local health-care workers in the optimal management of critically ill children in the Republic of Georgia.  Over a period of 18 months and four visits to the country, the authors  worked with Georgian pediatric critical care leadership to complete the following tasks: (1) survey health-care needs within the Republic of Georgia, (2) present representative PFCCS lectures and simulation scenarios to evaluate interest and obtain “buy-in” from key  stakeholders throughout the Georgian educational infrastructure, and (3) identify PFCCS instructor candidates.  Georgian PFCCS instructor training included the following steps:  (1) US PFCCS consultant and content experts presented PFCCS course to Georgian instructor candidates. (2) Simulation learning principles were taught and basic equipment was acquired. (3) Instructor candidates presented PFCCS to Georgian learners, mentored by PFCCS course consultants. Objective evaluation and debriefing with instructor candidates concluded each visit. Between training visits Georgian instructors translated PFCCS  slides to the Georgian language. Six candidates were identified and completed PFCCS instructor training. These Georgian instructors independently presented the PFCCS course to 15 Georgian medical students. | 4 training events over 18 months | Effective, sustainable training programme was established.  Student test scores improved significantly from pretest  results (n = 14) (pretest: 38.7 ± 7 vs. posttest 62.7 ± 6, p < 0.05). A Likert-type scale of 1  to 5 (1 = not useful or effective, 5 = extremely useful or effective) was used to evaluate each student’s perception regarding (1) relevance of course content to clinical work students rated as median (IQR): (a) relevance of PFCCS content to clinical work, 5 (4–5); (b) effectiveness  of lecture delivery, 4 (3–4); and (c) value of skill stations for clinical practice, 5 (4–5).  Additionally, the mean (±SD) responses were 4.6 (±0.5), 3.7 (±0.6), and 4.5 (±0.6), respectively. | Training local PFCCS instructors within an international environment is an effective method for establishing a uniform, reproducible, and sustainable approach to educating  health-care providers in the fundamentals of pediatric critical care. Future collaborations will evaluate the clinical impact of PFCCS throughout the Georgian health-care system. |
| 18 | Day et al. (2023) | Fostering Success and Promoting Professional Development of Clinician Educator Mentees: A Workshop for Mentors | Qualitative | Clinician educators trained on mentorship.  N=26 | The goal of academic mentoring relationships is to enable the mentee to identify/achieve professional advancement.  Although mentors of clinician educators (CEs) must understand the criteria for successful career advancement, few have received formal CE mentor training.  Hence, the National Research Mentoring Network convened an expert panel to develop a 90-minute module for training CE mentors. This module included individual development plans, case studies involving challenges for CE faculty, and examples of the broadened scope of scholarly activity. The workshop was delivered to 26 participants across four institutions and evaluated by a retrospective pre/post survey. | 90-minute module | Improvement in ability as a clinician educator, most notably setting and aligning expectations, along with setting career goals for mentees. | This module trains CE mentors using an interactive and collective problem-solving approach. Workshop participants better  defined demonstrable markers for CE progression with potential to impact tailored guidance for mentees. |
| 19 | de Dios et al. (2013) | The development of a diversity mentoring program for faculty and trainees: A program at the Brown Clinical Psychology Training Consortium | Qualitative | Implementation of a diversity mentorship programme at Brown University  N=14 | This paper outlines the early development and pilot implementation of a diversity mentorship program at Brown University. In an effort to inform and guide future diversity programs, we discuss the challenges faced in creating the program, the successes experienced during the first year, and the future directions undertaken as a means for improving the program. | One-off training | Overall, mentors reported being satisfied with the program and their match, and stated that they had developed good rapport with their mentees. Majority of mentors also felt they became more effective in handling diversity related issues. | The diversity mentoring program committee considers the program’s ongoing development successful and the program implementation to be both feasible and acceptable. Most notably, the vast majority of our participants rated their experience in a highly favorable manner and expressed a desire to continue their current mentoring relationship. |
| 20 | Donato and George (2012) | A blueprint for implementation of a structured portfolio in an internal medicine residency | Qualitative | Creation of a learning environment to support portfolio development.  N=7 | The authors review the  components necessary to successfully build and maintain a robust portfolio  learning environment in a graduate  medical education setting. | Focus group session | Training mentors is an important component in guiding effective portfolio development. These include  gaining staff acceptance, staging implementation, enhancing learner  participation, training mentors, choosing paper versus electronic formats, and  selecting evaluation methods. | Their blueprint for implementing a portfolio is  informed by their five-year experience with a portfolio rollout in one internal  medicine residency, from 2006 to 2011. |
| 21 | Eardley et al. (2020) | European Society of Coloproctology Colorectal Robotic Surgery Training for the Trainers Course - the first pilot experience | Qualitative | Robot-assisted colorectal surgery train-the-trainer course to improve training curriculum.  N=8 | Currently, there is no established colorectal speci- fic robotic surgery Train the Trainer (TTT) course. The aim was to develop and evaluate such a course which can then be further developed to be incorporated within the planned European Society of Coloproctology (ESCP)/European School of Coloproctology (ESC) robotic colorectal surgery training curriculum.  After identifying the need for such a course within a training programme, the course was developed by a subgroup of the ESCP/ESC. A scoping literature review was performed and the content and materials for the course were developed by a team consisting of two gastroenterologists with a combined experience of 30 years of facilitating TTT courses, a robotic surgeon and proctor with laparoscopic TTT faculty experience and experienced robotic and laparoscopic colorectal trainers. The course was evaluated by asking delegates to complete pre- and post-course questionnaires. | 2-day course | There were eight delegates on the course from across Europe. Delegates increased their knowledge of each of the course learning objectives and identified learning points in order to change practice. The feedback from the delegates of the course was positive across several areas and all felt that they had achieved their own personal objectives in attending the course. | This pilot robotic colorectal TTT course has achieved its aim and demonstrated many positives. There is a need for such a course and the evaluation processes have provided opportunities for reflection, which will allow the development/tailoring of future robotic colorectal TTT courses to help develop robotic training further. |
| 22 | Ehrich et al. (2004) | Formal Mentoring Programs in Education and Other Professions: A Review of the Literature | Systematic Review | Review on mentoring across disciplines | This paper draws upon a structured  analysis of over 300 research-based papers on mentoring across three discipline areas in an attempt to make more valid inferences about the nature and outcomes of mentoring. | NIL | The paper begins  by reporting on the findings compiled from a database of research papers from educational contexts.These research-based papers are examined to determine the positive and more  problematic outcomes of mentoring for the mentor, mentee and the organization. A discussion of the findings from two other databases, namely, 151 research-based papers from business  contexts and 82 papers from medical contexts, is provided and commonalities across the three databases are highlighted. | The paper concludes with a discussion of key issues that  administrators responsible for establishing mentoring programs should consider to maximise the experience of mentoring for all stakeholders. |
| 23 | Feldman et al. (2009) | Training the next generation of research mentors: the University of California, San Francisco, Clinical & Translational Science Institute Mentor Development Program | Qualitative | Mentor Development Programme established at UCSF.  N=29 | Mentoring is a critical component of career development and success for clinical translational science research faculty. Yet few programs  train faculty in mentoring skills.  The authors describe outcomes from the first two faculty cohorts who completed a Mentor Development Program (MDP) at UCSF. Eligibility includes having dedicated research time, expertise in a scientific area and a desire to be a lead research mentor. A post-MDP survey measured the program’s impact on enhancement of five key mentoring skills, change in the Mentors-in-Training  (MIT) self-rated importance of being a mentor to their career satisfaction, and overall confi dence in their mentoring skills. Since 2007,  29 MITs participated in and 26 completed the MDP. Only 15% of the MITs reported any previous mentor training. | Monthly half-day seminars | Being a mentor contributes towards career satisfaction.  Overall, 96% of MITs felt that participation in the MDP helped them to become better mentors. A majority reported a significant increase in confidence in mentoring skills and most reported an increased understanding of important mentoring issues at UCSF. MITs reported increased confidence in overall and specific mentoring skills after completion of the MDP. | The MDP can serve as a model for other institutions to  develop the next generation of clinical-translational research mentors. |
| 24 | Feldman et al. (2012) | A mentor development program for clinical translational science faculty leads to sustained, improved confidence in mentoring skills | Qualitative | Mentor Development Programme developed for clinical and translational faculty.  N=38 | Mentorship is crucial for academic productivity and advancement for clinical and translational (CT) science faculty. However, little  is known about the long-term effects of mentor training programs."The University of California, San Francisco (UCSF), Clinical and  Translational Science Institute launched a Mentor Development Program (MDP) in 2007 for CT faculty." The authors report on an evaluation of the first three cohorts of graduates from the MDP. In 2010, all Mentors in Training (MITs) who completed the MDP from 2007  to 2009 ( n = 38) were asked to complete an evaluation of their mentoring skills and knowledge; all MITs (100%) completed the  evaluation. | Monthly seminars | Two-thirds of MDP graduates reported that they often apply knowledge, attitudes, or skills obtained in the MDP to their  mentoring. Nearly all graduates (97%) considered being a mentor important to their career satisfaction. Graduates were also asked  about the MDP’s impact on specific mentoring skills; 95% agreed that the MDP helped them to become a better mentor and to  focus their mentoring goals. The authors also describe a number of new initiatives to support mentoring at UCSF that have evolved from  the MDP. | To our knowledge, this is the first evaluation of the long-term impact of a mentor training program for CT researchers. |
| 25 | Fornari et al. (2014) | Mentoring program design and implementation in new medical schools | Qualitative | Review of mentoring programmes across medical schools. | Mentoring is considered a valuable component of undergraduate medical education with a variety of  programs at established medical schools. This study presents how new medical schools have set up mentoring programs as they have developed their curricula.  Administrators from 14 US medical schools established since 2006 were surveyed regarding the  structure and implementation of their mentoring programs. | NIL | The majority of new medical schools had mentoring programs that varied in structure and implementation. Although the programs were viewed as valuable at each institution, challenges when creating  and implementing mentoring programs in new medical schools included time constraints for faculty and  students, and lack of financial and professional incentives for faculty. | Similar to established medical schools, there was little uniformity among mentoring programs at  new medical schools, likely reflecting differences in curriculum and program goals. Outcome measures are  needed to determine whether a best practice for mentoring can be established. |
| 26 | Freeman (2017) | Towards effective mentoring in general practice | Qualitative | Mentor project for professional development and increasing well-being among general practitioners  N=25 | In South Thames, a three-year feasibility study was designed to  explore the potential contribution of mentors in furthering professional  development and increasing a sense of well-being among general practitioners. The study led to the establishment of an ongoing mentor project as a South Thames (West)  Regional initiative, funded by the Postgraduate Dean of General  Practice.This paper outlines the project’s structure and administration.The concept of the holistic mentor model, together  with a strategy (the reflective cycle) for purposeful intervention, is also described. | 2 months of training | The findings from the evaluation of the study  illustrate the response of doctors to their mentor training, their early experience of taking on the role of mentor, and their  reporting of the experience of those being mentored (the  mentees). | The issues relevant to the future of mentoring are  commented on, to inform and encourage further discussion. |
| 27 | Gandhi et al. (2014) | Development and implementation of a workshop to enhance the effectiveness of mentors working with diverse mentees in HIV research | Qualitative | Development of a mentor training workshop for HIV researchers  N=26 | The authors describe the  development and implementation of a 2-day intensive workshop to train mid-level and senior-level investigators conducting HIV-related clinical and translational research across multiple academic institutions on more effective mentoring, with an emphasis on techniques to foster mentees of diversity. The workshop was focused on training mentors in techniques designed to improve the effectiveness of the mentor–mentee relationship, and included didactic presentations, interactive discussions, and small-group problem-based learning activities.  Mid-level or senior-level faculty involved or planning to be involved in significant mentorship activities related to HIV research were eligible. Surveys and formal actions plans allowed for workshop evaluation and laid the groundwork for subsequent workshops. Twenty-six faculty from 16 U.S.-based institutions participated, with good representation across discipline, gender, and race/ethnicity. | 2-day intensive workshop | The sessions were highly rated and discussions and evaluations revealed important barriers and facilitators to mentoring, challenges and solutions related to mentoring mentees from diverse backgrounds, and specific tools to enhance mentoring effectiveness. The Mentoring the Mentors training program for HIV researchers focusing on early career investigators of diversity was the first of its kind and was well attended, was rated highly, and provided guidance for improving the  program in the future. | This training program fills an important gap in the HIV researcher community and offers guidance for training mentors interested in diversity issues in settings outside of HIV. |
| 28 | Gandhi and Johnson (2016) | Creating More Effective Mentors: Mentoring the Mentor | Qualitative | Development of mentor training programmes for HIV biomedical researchers.  N=67 | Given the diversity of those affected by HIV, increasing diversity in the HIV biomedical research workforce is imperative. A growing body of empirical and experimental  evidence supports the importance of strong mentorship in the development and success of trainees and early career investigators in academic research settings, especially for mentees of diversity. Often missing from this discussion is the need for robust mentoring training programs to ensure that mentors are trained in best practices on the tools and techniques of mentoring. Recent  experimental evidence shows improvement in mentor and mentee perceptions of mentor’s  competency after structured and formalized training on best practices in mentoring.  Hence, the authors developed a 2-day “Mentoring the Mentors” workshop at UCSF to train mid-level  and senior HIV researchers from around the country (recruited mainly from Centers for AIDS Research (CFARs)) on best practices, tools and techniques of effective mentoring. The workshop content was designed using principles of Social Cognitive Career Theory (SCCT) and include training specific to working with early career investigators from underrepresented groups, including training on unconscious bias, microaggressions, and diversity supplements. The workshop has been held 3 times (September 2012, October 2013 and May 2015) with plans for annual training. Mentoring competency was measured using a validated tool before and after each  workshop. | 2-day workshop on best practices, tools, and techniques. | Mentoring competency skills in six domains of mentoring -specifically effective  communication, aligning expectations, assessing understanding, fostering independence, addressing diversity and promoting development - all improved as assessed by a validated  measurement tool for participants pre- and-post the “Mentoring the Mentors” training workshops.  Qualitative assessments indicated a greater awareness of the micro-insults and unconscious bias  experienced by mentees of diversity and a commitment to improve awareness and mitigate these effects via the mentor-mentee relationship. | The developed “Mentoring the Mentors” workshop for HIV researchers/mentors offers a  formal and structured curriculum on best practices, tools and techniques of effective mentoring,  and methods to mitigate unconscious bias in the mentoring relationship and at the institutional level with mentees of diversity. The authors found quantitative and qualitative improvements in mentoring skills as assessed by self-report by participants after each workshop and plan additional programs  with longitudinal longer-term assessments focused on objective mentee outcomes (grants, papers, academic retention). Mentoring training can improve mentoring skills and are likely to improve  outcomes for optimally-mentored mentees. |
| 29 | Harris et al. (2022) | Empowering Frontline Primary Healthcare Workers in a Global Health Partnership Training of Trainers Intervention to Strengthen the Prevention and Control of Cardiovascular Disease in Mozambique | Feasibility study | Training of primary care physicians in Mozambique to educate others on cardiovascular disease and its prevention.  N=60 | Unpreparedness of health professionals to address non-communicable diseases (NCD) at peripheral health facilities is a critical health system challenge in Mozambique. To address this weakness and decentralize NCD care, training of the primary care workforce is needed. The authors describe their experience in the design and  implementation of a cascade training of trainers (ToT) intervention to strengthen the prevention and control of cardiovascular disease.  Between October 2018 and March 2020 a multidisciplinary global technical partnership was used to train frontline primary care health professionals from a resource-poor suburban setting in Maputo, Mozambique. Following engagement with local policy makers, clinicians, and academics, core training materials were developed, and a ToT cascade was implemented, supported by an on-site pilot clinic. Knowledge  and confidence acquisition by participants and new local trainers were assessed using pre- and post-training surveys, while trainees and trainers completed further  evaluation surveys at the end of the program. | Three workshop sessions | Three ToT workshops trained 60 mixed cadre healthcare workers in assessment,  diagnosis and management of hypertension, diabetes, and cardiovascular risk; of these,  11 became new local trainers. Mean pre- and post-test scores improved in all three  training workshops (53% to 90%, 59% to 78%, and 58% to 74% respectively). New  local trainers were highly rated by their trainees and reported increased confidence as  trainers (mean Likert scale 3.0/5 pre-training to 4.8/5 post-training).  Healthcare needs are better met through cascade intervention. | This global health partnership delivered interprofessional training with good knowledge acquisition and increased self-reported confidence. Intensive local supervision and hands-on training empowered a new cohort of trainers to strengthen the prevention and control of cardiovascular disease and is likely to improve coordination and integration at primary care level as well as support the national scale up of NCD care delivery. |
| 30 | Hill et al. (2022) | The Nature and Evolution of the Mentoring Relationship in Academic Health Centers | Review | Review of the qualities that are most critical in developing a successful mentor-mentee relationship, as well as the evolution of the longitudinal nature of it | This article reviews the qualities that are most critical in developing a successful mentoring relationship, the longitudinal nature of this relationship, common problems that arise, and the potential rewards that exist for each person involved in the relationship. | NIL | An effective mentor is most commonly described as supportive, has integrity, is a role model and a good communicator with effective messaging. The mentor also has to have compatible traits with mentees, is accessible to learners and sets goals and expectations.  Mentors need to be equipped to know how to handle mismatch of personalities, as well as maintain professional boundaries and protect confidentiality. They need to be conscious of their inherent biases too. | Good mentoring is a facilitative, developmental and positive process which requires adequate time, intentional discourse, mutual respect, and a willingness to support the relationship. There are clear benefits for both mentees and mentors who work together to develop this type of relationship. Most successful relationships include individuals that demonstrate key characteristics as highlighted in this paper. The mentoring relationship is not static; rather, it is a dynamic process that evolves over time. It is as unique as the people who engage in this worthwhile venture. Dedicated individuals, who are intentional in their interactions with one another, will surely gain benefits in their careers and may even develop an enduring partnership through their experience in mentoring. Common pitfalls and problems as well as ways to address such problems were also included here along with prevention and intervention strategies. In general, personal reflection about the mentor relationship both before initiating it and throughout its course, by both the mentor and mentee, will contribute to a robust, growth-oriented relationship that meets expectations and achieves targeted goals. |
| 31 | Holmboe et al. (2011) | Faculty development in assessment: the missing link in competency-based medical education | Qualitative | Suggestions for development of faculty | Substantial evidence suggests,  that current faculty are insufficiently  prepared to meet the needs of an increasingly diverse and aging population. There are inadequacies across both the traditional competencies of medical  knowledge, clinical skills, and  professionalism and the newer  competencies of evidence-based  practice, quality improvement,  interdisciplinary teamwork, and systems. Hence, in this article, the authors outline the current challenges and provide suggestions on where faculty development efforts should be focused and how such an initiative might be accomplished. The public, patients, and trainees need the medical education enterprise to improve training and outcomes now. | NIL | The faculty who participate in educational training activities report (1) high levels of satisfaction, (2) positive changes in their attitudes, (3) increased understanding of educational principles and teaching skills, (4) changes in behavior as noted by their students, and (5) greater involvement in teaching. This study also noted that success factors for faculty development include incorporation of feedback in the training, active learning, effective relationships with peers and colleagues, and use of diverse teaching approaches. | Teaching practices must change to accommodate an increasingly diverse and ageing population. |
| 32 | Johnson et al. (2010) | An innovative program to train health sciences researchers to be effective clinical and translational research mentors | Qualitative | Mentor Development Programme (MDP) for mid-level investigators to be more effective mentors. | There are numerous substantive barriers to effective mentoring, which include a lack of training in how to be a mentor, lack of time and structural and financial  support for mentoring, and competing personal, administrative and clinical demands.  Hence, the authors describe an innovative program, the University of California, San Francisco (UCSF)  Mentor Development Program (MDP), established in 2006 and designed to train mid-career  academic health sciences researchers to be more effective as clinical and translational research  mentors. Using a framework for presenting innovations in academic research, they present the  rationale, design, implementation, and mechanisms being used to evaluate and sustain the MDP. | 10 seminars, 2 per month | Specific details of the objectives and content of the MDP sessions are provided as well as  evaluation criteria and a link to specific curriculum materials. Objectives, content and evaluation of MDP outcomes are presented | Using a framework for presenting innovations in academic research, the authors presented the rationale, design, implementation, and  mechanisms to evaluate and sustain the resulting program, the UCSF Mentor Development  Program (MDP). |
| 33 | Johnson, Gandhi (2015) | A mentor training program improves mentoring competency for researchers working with early-career investigators from underrepresented backgrounds | Qualitative | Mentoring-the-mentor workshop for HIV-related clinical researchers.  N=34 | There are limited programs to train faculty to become more effective  mentors, and the few that exist have a dearth of empirical support of their impact. Hence, this paper aims to address that gap and document its impact.  In 2013, the authors recruited 34 faculty from across the US engaged in HIV-related clinical research to  participate in a 2-day Mentoring the Mentors workshop. The workshop included didactic and interactive content focused on a range of topics, such as mentor–mentee communication, leadership styles, emotional intelligence, understanding the impact of diversity  (unconscious bias, microaggressions, discrimination, tokenism) for mentees, and specific  tools and techniques for effective mentoring. | 2-day workshop | Pre- and post-workshop online evaluations documented high rates of satisfaction with the program and statistically significant improvements in self-appraised mentoring skills (e.g. addressing diversity in mentoring, communication with mentees, aligning mentor–mentee expectations), as assessed via a  validated mentoring competency tool.. | This is the first mentoring training program focused  on enhancing mentors’ abilities to nurture investigators of diversity, filling an important gap, and evaluation results offer support for its effectiveness. Results suggest a need for  refinement and expansion of the program and for more comprehensive, long-term evaluation of distal mentoring outcomes for those who participate in the program. |
| 34 | Karia et al. (2020) | Peer teacher training (PTT) in action | Feasibility study | Peer-teacher-training to develop skills in undergraduate medical students  N=20 | This study evaluated the implementation of a PTT programme adopted from the Medical School at the University of Sydney in 2016. The pro- gramme was adopted to support the development of skills in UK medical undergraduates. The training was accessed by students studying at the University of Leicester in 2017 and 2018.  The course was evaluated using mixed methods. Students completed pre- and post-course self-rated question- naires. Semi-structured focus groups were conducted after each course to investigate students’ perceptions. | 6 modules, one online and the rest in a day-long session. | Twenty fourth-year medical students attended the PTT. Students reported improve- ments in their understanding of educational principles, confi- dence in teaching and feedback skills, and an increased likeli- hood of volunteering to teach. Focus groups revealed potential benefits for future patient interaction and preparation for educator roles. | The PTT provided an effective approach for medical students to gain perceived competence and confidence in teaching and giving feedback. Students reported an increased propen- sity for teaching when qualified. Students with lower self- reported teaching ability reported greater improvements following the course. Students reported unanticipated per- ceived benefits for improving patient education and obtaining teaching accreditation for their portfolio and future clinical roles. They perceived potential value if the training were to be made interprofessional on future courses. Furthermore, this study demonstrates the transferability of the PTT programme across university settings. |
| 35 | Kashiwagi et al. (2013) | Mentoring programs for physicians in academic medicine: a systematic review | Systematic review | Review of mentoring programmes for practicing physicians | Mentoring is vital to professional development in the field of medicine,  influencing career choice and faculty retention; thus, the authors reviewed  mentoring programs for physicians and aimed to identify key components that contribute to these programs’ success.  The authors searched the MEDLINE, EMBASE, and Scopus databases for articles from January 2000 through May  2011 that described mentoring programs for practicing physicians. The authors reviewed 16 articles, describing 18 programs, extracting program objectives, components, and outcomes. They synthesized findings to determine key  elements of successful programs. | NIL | All of the programs described in the articles focused on academic physicians.  The authors identified seven mentoring models: dyad, peer, facilitated peer, speed, functional, group, and distance. The dyad model was most common. The authors identified seven potential  components of a formal mentoring program: mentor preparation, planning  committees, mentor–mentee contracts, mentor–mentee pairing, mentoring activities, formal curricula, and program  funding. Of these, the formation of mentor–mentee pairs received the most  attention in published reports. Mentees favored choosing their own mentors; mentors and mentees alike valued  protected time. One barrier to program development was limited resources. Written agreements were important to  set limits and encourage accountability to the mentoring relationship. Program  evaluation was primarily subjective, using locally developed surveys. No programs reported long-term results | The authors identified key program elements that could contribute to successful physician mentoring. Future  research might further clarify the use of these elements and employ standardized  evaluation methods to determine the long-term effects of mentoring |
| 36 | Kaul et al. (2020) | COLONOSCOPY SKILLS AND TRAIN THE TRAINER COURSES FOR PEDIATRIC ENDOSCOPY: NORTH AMERICAN EXPERIENCE | Feasibility study | Simulation was employed in teaching pediatric endoscopists through a train-the-trainer framework.  Colonoscopy course: N=66  Train-the-trainer course: N=37 | Simulation is increasingly being recognized as an important modality to not only train colonoscopy skills but also to improve supervisors’ abilities to teach endoscopy effectively. The authors hypothesize that endoscopy training can be enhanced by using simulation to teach endoscopists standardized colonoscopy skills and an endoscopy training framework. The paper aims to evaluate endoscopists’ experiences  with simulation-based Colonoscopy Skills and Train the Trainer courses tailored specifically to pediatric endoscopy.  Six simulation-based Colonoscopy Skills courses and three Train the Trainer courses were offered at an annual pediatric conference for pediatric gastroenterology (GI) faculty and fellows in 2017, 2018, and 2019. The hands-on courses were led by physicians with expertise in endoscopy education. Surveys were completed before and after each course to evaluate participants’ prior experience and the perceived impact of the courses. Respondents rated their level of agreement with each statement on a 1 (strongly disagree) to 5 (strongly agree) point scale. Responses were analyzed using descriptive statistics. | 6 simulation-based colonoscopy courses and 3 train-the-trainer courses conducted. | There was a significant reported improvement in both knowledge and skills in colonoscopy post-course.  Participants also reported an improved understanding of the “Preparation-Training-Wrap-Up” endoscopy training framework, ability to align goals of the trainer and trainee, use of standardized training language, performance enhancing feedback, improved conscious competence, and endoscopy room set-up. | Simulation-based Colonoscopy Skills and Train the Trainer courses improved pediatric endoscopists’ perceived knowledge and skills related to both performing and  training endoscopy. Future prospective studies to assess course impact are required. |
| 37 | Keyser et al. (2008) | Advancing institutional efforts to support research mentorship: a conceptual framework and self-assessment tool | Review | Review of methods for advancing efforts in supporting research mentorship | The purpose of this article is to assist institutions in advancing their efforts to support research mentorship. The authors begin by describing how institutions can shape the key domains  of research mentorship: (1) the criteria for selecting mentors, (2) incentives for motivating faculty to serve effectively as mentors, (3) factors that facilitate the mentor–mentee relationship, (4)  factors that strengthen a mentee’s  ability to conduct research responsibly, and (5) factors that contribute to the professional development of both mentees and mentors.  On the basis of a conceptual analysis of these domains as currently documented in the literature, as well as their collective  experience examining mentoring  programs at a range of academic  medicine institutions and departments, | NIL | The authors provide a framework that leaders of institutions and/or departments  can adapt for use as a tool to document and monitor policies for guiding the mentorship process, the programs/activities  through which these policies are implemented, and the structures that are responsible for maintaining policies and  implementing programs. The authors provide an example of how one hypothetical institution might use the  self-assessment tool to track its policies, programs, and structures across the key  domains of research mentorship and, on  the basis of this information, identify a range of potential actions to strengthen its  research mentoring efforts. | The authors  conclude with a brief discussion of the  limitations of the self-assessment tool, the  potential drawbacks and benefits of the  overall approach, and proposed next steps  for research in this area. |
| 38 | Kirresh et al. (2011) | A framework to establish a mentoring programme in surgery | Qualitative | Review of key features of a surgical mentoring programme and establishment of an effective mentoring programme | Mentoring programmes help to facilitate the process of continuous professional development in surgery,  providing an organizational structure around a mentor–mentee  relationship which helps to develop the mentee. The lack of guidelines outlining how to set up such mentoring programmes, the fragmented inter-relationships of existing schemes and the lack of a unified strategy for their implementation are obstacles to the creation of such initiatives within many surgical departments. Hence, the authors draw upon previous research, the experiences of  certain authors and their own reflections to identify the key  features of a surgical mentoring programme. | NIL | A ten-step process for formal mentoring programme development in surgery is described. Mentoring programmes are difficult to support due to opportunity cost of a surgeon’s revenue. | This outline may improve the delivery and  effectiveness of mentoring programmes, which may ultimately enhance surgical training and hence quality of  patient care. |
| 39 | Kirsch et al. (2018) | Career-Focused Mentoring for Early-Career Clinician Educators in Academic General Internal Medicine | Qualitative | Establishment of a formal early career mentoring programme in general internal medicine  N=8 | Clinician educators face challenges in developing academic careers and advancing in rank. They may have limited knowledge and skills in curriculum development and research methods or in pedagogy and medical education theory. Most clinician educator effort is spent balancing competing demands of clinical productivity and teaching, which limits time for fulfilling scholarship expectations. This article describes the value of a mentoring program aimed at ameliorating such challenges for early-career clinician educators. This article describes an approach in an academic general internal medicine division to ensure more equitable access to mentors by establishing a semi-structured faculty career mentoring program (CMP), and empower faculty participants in the CMP—via scheduled mentoring discussions, individual development plans (IDPs), and professional development programming—to become more proactive managers of their academic careers and productive scholars. The objective of this article is to report on a model program with the intent of proving an example for structured early-career mentoring for nontenure-track faculty. | 90-minute training session.  Mentors met annually to discuss challenges and strategies | It was reported that there was increased efficacy for career management, increased personal drive and reduced burnout.  Improvements were also seen in mentee overall satisfaction with mentoring received (past 24 months), satisfaction with career mentoring in 2 specific areas (work-life balance, overcoming professional challenges), understanding of mentee roles, and perceived importance of mentoring for career development and retention | In conclusion, the results suggest that a semi-structured CMP can have desirable outcomes for clinician educator faculty, including increased self-efficacy for career management, more positive mentoring attitudes, and greater scholarly productivity. Division-level oversight helps ensure access to mentors, who play facilitative roles helping clinician educators align their interests/activities with institutional expectations for advancement. |
| 40 | Lewellen-Williams et al. (2006) | The POD: A New Model for Mentoring Underrepresented Minority Faculty | Qualitative | Peer-Onsite-Distance model for achieving mentee’s career goals and the resources needed for development  N=19 | This paper explores the Peer-Onsite-Distance (POD) model,  developed in 2002 by the authors and introduced at the College of Medicine at the University of Arkansas for Medical Sciences, is a targeted, multilevel mentoring prototype that is built on a  solid research foundation and tailored to the unique needs of URM medical school faculty. The mentee’s individual needs for guidance related to career goals,  resources, and the content and  interaction skills that are known to be critical to successful academic careers are targeted for development. | Basic or refresher courses for mentors | The multilevel approach provides a unique network of  peer and faculty mentors who provide site-specific career guidance. Also in the  network are leaders in their fields who can provide access to accurate information, cautions, predictions, and  announcements of future resources or potential restrictions in academic  medicine. Mentor commitments are clearly defined and time contributions  are maximized. | The POD model aims to  promote retention and advance the careers of URM faculty by wrapping them in a protective cushion of  interpersonal and intrapersonal support. It also improves effectiveness of leadership, advisory and teaching skills of mentors.  The flexibility of the design allows for adaptation to any institution’s unique  structure and mission. |
| 41 | Libby et al. (2016) | Grant Success for Early-Career Faculty in Patient-Oriented Research: Difference-in-Differences Evaluation of an Interdisciplinary Mentored Research Training Program | Qualitative | Clinical Faculty Scholars’ Programme, involving structured mentoring and structured training  N=25 | Since 2004, the Clinical Faculty Scholars Program (CFSP) at the University of Colorado Anschutz Medical Campus has provided intensive interdisciplinary mentoring and structured training for early-career clinical faculty from multiple disciplines conducting patientoriented clinical and outcomes research. This study evaluated the two-year program’s effects by comparing grant outcomes for CFSP participants and a matched comparison cohort of other junior faculty.  Using 2000–2011 institutional grant and employment data, a cohort of 25 scholars was matched to a cohort of 125 comparison faculty (using time in rank and pre-period grant dollars awarded). A quasi-experimental difference-in-differences design was used to identify the CFSP  effect on grant outcomes. Grant outcomes were measured by counts and dollars of grant proposals  and awards as principal investigator. Outcomes were compared within cohorts over time (pre- vs post-period) and across cohorts. | Research training of mentees by qualified mentors | From pre-to post-period, mean annual counts and dollars of grant awards increased  significantly for both cohorts, but mean annual dollars increased significantly more for the CFSP than for the comparison cohort (delta $83,427 vs. $27,343, P < .01). Mean annual counts of grant  proposals also increased significantly more for the CFSP than for the comparison cohort: 0.42 to 2.34 (delta 1.91) vs. 0.77 to 1.07 (delta 0.30), P < .01. | Institutional investment in mentored research training for junior faculty provided significant grant award gains that began after 1 year of CFSP participation and persisted over time.  The CFSP is a financially sustainable program with effects that are predictable, significant, and enduring. |
| 42 | Luo et al. (2021) | Costs of a Train-the-Trainer Program to Teach Primary Care Faculty Mental Health Care | Qualitative | Proposition of train-the-trainer programme to educate medical school faculty on mental health. | This paper aims to estimate the direct cost of implementing an evidence-based Train-the-Trainer (3T) program to disseminate mental health training to allopathic medical school faculty; once trained, faculty can teach a much-enhanced curriculum of mental health care to medical students and residents.  A combination of published standardized unit costs and  an activity-based costing approach is used to estimate the direct costs  (labor and nonlabor) for implementing the 3T program. | NIL | The estimated direct cost of implementing the 3T program  at one prototypical school, including the 12-month start-up period (1.1 million) and 18-month rollout period (8.6 million), is ∼9.7 million dollars. | Wide outreach to all United States allopathic medical schools can be achieved with train-the-trainer programmes.  Successfully adopted in all US allopathic medical schools, the 3T program will provide over 3800 attitudinally competent and mental health skills-qualified primary care faculty members. They would then be available to train nearly 100,000 medical students per year and 55,000 primary care residents to be as competent in basic mental health care as in medical care. This 3T program will begin to meet the needs each year for the millions of adults with major mental disorders that now are largely unrecognized and untreated. |
| 43 | McCullough et al. (2015) | How can clinician-educator training programs be optimized to match clinician motivations and concerns? | Review | Review of motivations and barriers for clinician-educators to improve training programmes. | The goal of this study was to review the literature to identify motivations and perceived barriers to clinician-educators, which in turn will improve clinician-educator training programs to better align with clinician-educator needs and concerns.  Review of medical education literature using the terms “attitudes”, “motivations”, “physicians”, “teaching”, and “undergraduate medical education” resulted in identification of key themes revealing the primary motivations and barriers involved in physicians teaching undergraduate medical students. | NIL | A synthesis of articles revealed that physicians are primarily motivated to teach undergraduate students for intrinsic reasons. To a lesser extent, physicians are motivated to teach for extrinsic reasons, such as rewards or recognition. The key barriers deterring physicians from teaching medical students included: decreased productivity, lack of compensation, increased length of the working day, patient concerns/ethical issues, and lack of confidence in their own ability. | Optimization of clinician-educator training programs should address, amongst other factors, time management concerns, appropriate academic recognition for teaching service, and confidence in teaching ability. Addressing these issues may increase the retention of clinicians who are active and proficient in medical education. |
| 44 | Ng et al. (2015) | Fostering mentorship for clinician-investigator trainees: overview and recommendations | Systematic review | Review of mentorship in Medicine | The Clinician Investigator Trainee Association of Canada/ Association des  cliniciens-chercheurs en formation du Canada (CITAC/ACCFC) recently published the first survey to assess factors contributing to trainee satisfaction. One key finding is that  increased level of mentorship strongly correlates with overall satisfaction; however, while 98% of respondents reported mentorship as important to success, more than 60% expressed some dissatisfaction with the mentorship received. To help address this discrepancy, we reviewed mentorship in academic medicine, focusing on clinician-investigator  trainees, and distilled a set of recommendations for mentors, mentees and institutions.  OVID and manual curation based on the search terms ‘mentorship’ AND ‘education, medical and research’ identified 198 articles. Two authours independently reviewed both titles and abstracts and narrowed them down to 75 articles, based on relevance to mentorship in academic medicine. Consensus resulted in the selection of 19 articles for detailed review. | NIL | Mentorship is beneficial at each training stage and is associated with greater research productivity, career retention and promotion.  Studies have identified the characteristics of good mentors, including the ability to ensure open communication, ability to maintain confidentiality and ability to ensure that there is no  mentor-mentee competition. | The characteristics of good mentees have been identified as the ability to take ownership of a project and the ability to build a network or  team of mentors. The literature has also identified the actions that institutions can take to  facilitate mentorship, which include mentor training and recognizing mentorship through  awards. |
| 45 | Nizeyimana et al. (2024) | Transfusion Camp Rwanda 2023: A train-the-trainer workshop establishing locally driven leadership in knowledge translation and sustainability in transfusion medicine education | Qualitative | Train-the-trainer programme in Rwanda on the topic of blood transfusion.  N=51 | Blood transfusion is performed daily in hospitals. Gaps exist between transfusion guidelines and day-to-day clinical care. These gaps are prevalent in resource-limited settings due to scarce continuing medical education.  Transfusion Camp Rwanda aims to bridge this gap by (1) delivering context- appropriate up-to-date education, (2) teaching participants how to independently deliver a case-based curriculum and (3) identifying strategies to promote change in transfusion practice in Rwanda.  In May 2023, a multidisciplinary team from Canada and  Rwanda carried out a Transfusion Camp train-the-trainer workshop for clinicians from  all five provinces in Rwanda. Participants attended in-person lectures, seminars and  workshop group discussions on the implementation of the Rwanda National Directives on Rational Use of Blood and Blood Components. Course feedback was based on the Kirkpatrick Model of Training and Evaluation. | Two workshop sessions, with didactic sessions and case-based small group seminars | Fifty-one physicians and laboratory technicians participated in the course.  Confidence in caring for patients based on transfusion guidelines was self-rated as  ‘excellent’ by 23% of participants before and 77% after, while 84% reported they  planned to teach Transfusion Camp to others and 100% responded that they will  apply course content to clinical practice. Workshop groups recommended strategies  to improve transfusion medicine practice in Rwanda in four domains: Communica-  tion, Institutional Approval, Practice Audits and Education. | Transfusion medicine education in Rwanda using a train-the-trainer approach was well-received by participants and allowed for a more detailed understanding of the local medical and educational environment. These observations can inform the further expansion of the Transfusion Camp Rwanda project. |
| 46 | Peralta et al. (2023) | “Train the Trainers” Program to Improve Knowledge, Attitudes and Perceptions About Organ Donation in the European Union and Neighbouring Countries: Pre- and Post- Data Analysis of the EUDONORGAN Project | Qualitative | Train-the-trainer programme for improving practice surrounding organ and tissue donation  N=79 | EUDONORGAN, a European Union-funded project to improve organ and tissue donation,  included a blended-based “Train the Trainers” program, which was implemented with the support of an international consortium from Croatia, Italy, Slovenia, and Spain.  The web-based training included seven modules for which medical aspects, educational tips, and practical activities were scored using a 5-point Likert scale. | 7-module online training, followed by face-to-face training | The overall mean scores of  satisfaction were higher than 4 for each module, without significant differences between  HCPs and OKPs. In the face-to-face training survey similar scores above 4 were obtained  for most items. Knowledge acquisition improved significantly in both HCPs and OKPs, as  well as in transplant/donor coordinators, medical doctors, registered nurses,  anesthesiologists/intensivists, and intensive care nurses. Improvements in attitudes and  perceptions regarding organ donation were also observed, particularly among HCPs. In  the accomplishment of the learning process, a successful pass mark of 95% was  obtained. | The “Train the Trainers” program was associated with an improvement in  learning and attitudes of healthcare and non-healthcare professionals for the benefit of  organ and tissue donation. |
| 47 | Pfund et al. (2014) | Training mentors of clinical and translational research scholars: a randomized controlled trial | Randomised controlled trial | Investigating the effect of a competency-based, formal mentor training programmes on mentoring skills  N=144 | This paper aims to  determine whether a structured  mentoring curriculum improves research mentoring skills.  The authors conducted a randomised controlled trial (RCT) at 16 academic health centres (June 2010 to July 2011). Faculty mentors of trainees who were conducting clinical/translational research ≥50% of the time were eligible.  The intervention was an eight-hour,  case-based curriculum focused on six mentoring competencies. | 8-hour case-based curriculum session | Scores measured by Mentoring Competency Assessment were higher for formally trained mentors compared compared to those without formal training.  The primary outcome was the change in mentors’ self-reported pretest to posttest composite scores on the Mentoring Competency Assessment (MCA). Secondary outcomes included changes in the following: mentors’ awareness as measured by their self-reported retrospective change in MCA scores, mentees’ ratings of their mentors’ competency as measured by MCA scores, and mentoring behaviours as reported by mentors and their mentees. | This RCT demonstrates that a competency based  research mentor training program  can improve mentors’ skills. |
| 48 | Pfund et al. (2016) | Defining Attributes and Metrics of Effective Research Mentoring Relationships | Qualitative | Review of attributes of effective mentoring relationships | This paper proposes core attributes of effective mentoring relationships, as supported by the literature and suggested by theoretical models of academic persistence. In addition, both existing and developing metrics for measuring the effectiveness of  these attributes within mentoring relationships across diverse groups are presented, as well as preliminary data on these metrics from the authors’ work. | NIL | Characteristics of good mentors are identified. | Mentors must be knowledgeable about the institutional requirements and there is a lack of training in effective mentorship. |
| 49 | Phitayakorn et al. (2016) | Development and initial results of a mandatory department of surgery faculty mentoring pilot program | Qualitative | Development and results of a faculty mentorship programme in surgery  N=22 | Mentoring of junior faculty by senior faculty is an important part of promotion and/or tenure and enhanced job satisfaction. This study reports the development and results to date of a faculty mentorship program in surgery.  A departmental faculty mentoring program was implemented in July 2014 that consisted of both structured and informal meetings between junior faculty mentees and  assigned senior faculty mentors. All senior faculty mentors attended a brief mentor training session. Then an evidence-based mentorship instrument that  featured standardized metrics of academic success was developed. This instrument was completed by  each mentee, and then reviewed at the junior faculty’s annual career conference with their  division chief. A survey was distributed in July 2015 to assess junior faculty satisfaction  with the new mentorship program | 2-hour training | Over 75% of junior faculty members were very or somewhat satisfied with the mentorship program and would like to continue in the program. The best aspect of the program was the opportunity to meet with an accomplished surgeon outside their division. Opportunities to improve the program included better matching of mentor to mentee by disease or research focus. Interestingly, almost the entire junior faculty members tended to have at least two other mentors besides the mentor assigned to them in this program. In terms of program outcomes, junior faculty members agreed that the mentorship program improved their overall career plans and enhanced their involvement in professional organizations but has not yet helped with academic productivity, home and/or work balance, and overall job satisfaction. | A structured mentorship programme increases involvement and improves career planning of junior faculty. Mentor training increases understanding of the mentor role. |
| 50 | Schmidt et al. (2010) | Does the training of mentors increase the contact frequency and the quality of support in a portfolio-based teaching module? | Quantitative | Investigation of mentor training on mentoring relationship  N=14 | In the framework of the student’s evaluation of this training period it  was asked whether and what kind of effect the mentor training has had  on the contact frequency between mentor and student and whether it  affects how students experience the mentor’s support.  At the end of the training section the students evaluated the contact to their mentor with a structured question form. The questions on the contact frequency and how the students experienced the support  through the mentor were evaluated for the present study.  186 question forms were evaluated; 67 of them related to trained mentors. | One-day seminar | One year after the first training, the students rated the trained  mentor’s support significantly higher than the support by untrained mentors. There was a tendency noted, though not significant, for a higher contact frequency with the trained mentors. During the following three years, the measurable difference between the trained and untrained mentors regarding both items was not significant. In those years, a tendency towards a more intensive support of the students through all mentors was shown. | The evaluation results one year after the intervention imply  that trained mentors can intensify their support for the students without  requiring more time. The positive development of the evaluation results  for both mentor groups during the following three years can be interpreted  as a result of the process of exchange between trained and untrained  mentors and readjustment among staff. |
| 51 | Schweitzer et al. (2019) | Building a comprehensive mentoring academy for schools of health | Feasibility study | Development of a mentoring academy to increase uptake of mentoring roles  N=222 | A mentoring academy (MA) was developed for faculty across tracks (i.e., researchers, clinicians, educators) within a “school of health” encompassing schools of medicine and nursing. The program is anchored dually in a clinical and translational science center and a school of health. The structure includes the involvement of departmental and center mentoring directors to achieve widespread uptake and oversight. A fundamental resource provided by the MA includes providing workshops to enhance mentoring skills. Initiatives for junior faculty emphasize establishing and maintaining strong mentoring relationships and implementing individual development plans (IDPs) for career planning.  This paper presents self-report data on competency improvement from mentor workshops and data on resources and barriers identified by junior faculty (n = 222) in their IDPs. | Core module of 5 workshops, longitudinal programme | Mentors reported statistically significantly improved mentoring competency after workshop participation. Junior faculty most frequently identified mentors (61%) and collaborators (23%) as resources for goal attainment. | Top barriers to goal attainment included insufficient time and time-management issues (57%), funding limitations (18%), work–life balance issues (18%), including inadequate time for self-care and career development activities. |
| 52 | Selwa (2003) | Lessons in mentoring | Qualitative | Review of the role of mentoring on success in Medicine. | Sid Gilman, M.D., F.R.C.P., Chair of Neurology for 26 years at the University of Michigan, is well-recognized as a role model for mentors in neurology across the country. This paper reports the result of a survey of his former trainees on the valuable aspects of his mentoring style.  A review of the current mentoring literature, including suggested training programs for mentors, is also provided. | NIL | The highest correlation was between the likelihood of positively influencing career choice and the effectiveness of mentoring. The most commonly cited valuable mentoring attribute was the ability to serve as a role model, indicating that they would like to emulate both Dr Gilman’s skill as a neurologist and his personal qualities of integrity, honesty, and fairness (16 comments). | Formal education programmes for faculty improves role modeling of mentors to mentees and will allow mentors to achieve their full potential. |
| 53 | Shada et al. (2022) | Better than the real thing? Success of a virtual platform for an established "Train the Trainer" course | Qualitative | Train-the-trainer virtual course for laparoscopic colectomy  N=36 | The Society of American Gastrointestinal and Endoscopic Surgeons (SAGES) uses the Laparoscopic colectomy  Train the Trainer (Lapco TT) framework for standardization of instructor training for Hands-On surgical skills courses. The  curriculum focuses on teaching structure, skills deconstruction, trainer intervention framework, and performance enhancing  feedback. A halt in the in-person Lapco TT courses due to the Coronavirus Disease 2019 (COVID-19) pandemic necessitated  creation of a virtual alternative. This paper investigated the efectiveness of this virtual course.  Adaptation of the in-person Lapco TT course to the virtual format retained the majority of content as well as the 4:6 instructor-to-participant ratio. The virtual platform and simulators chosen allowed maximal interactivity and ease of use. After participating in the day and one half course, participants completed an 8-item post-course survey using a 5-point Likert scale related to the training experience. In addition, they had the opportunity to provide answers to several open-ended questions regarding the course. For the survey, frequency counts provided an assessment of each item. For the open questions, qualitative analysis included determination of themes for each question. Frequency counts of each theme provided quantitative analysis. | 1 online session per participant (6 sessions for 6 participants each) | Thirty-six total participants completed a Lapco TT virtual course (six sessions of six participants). Of this number,  32 participants completed post-course surveys and questions. All the participants completing the survey would very likely or defnitely (Likert scale 4, 5) recommend the course to a colleague and incorporate the teaching in their practice. The majority  of participants completing open-ended questions felt the virtual course format was efective; half thought that post-course follow-up would be useful. Technical concerns were an issue using the virtual format. | A virtual Lapco TT course is feasible and well received by participants. It presents a potentially more cost effective option to faculty development. |
| 54 | Shehu et al. (2023) | Train-the-trainers intervention for national capacity building in infection prevention and control for COVID-19 in Nigeria | Qualitative | Train-the-trainer course for prevention and control of COVID-19  N=61 | This study aims to describe the process and results of train-the-trainers as an  intervention for national capacity building in infection prevention and control for COVID-19 among healthcare workers in Nigeria.  Eight-hour sessions were held over three days with face-to-face instruction and practical hands-on experience in April 2020. A total of 61 healthcare workers participated across the six  geographic zones of Nigeria: North Central, North East, North West, South West, South East, and South South. The training included slide presentations, case-based scenarios, and practical hands-on sessions with plenary discussions. Pre- and post-test assessments were used to evaluate  knowledge of COVID-19, triage, and infection prevention and control among healthcare workers. | 8-hour workshops across 3 days | 69 % (42) of the participants were male 31 % (19) were female, and the majority  (67 %) were medical doctors. Others attending were nurses or health administrators. Of the 70% (26) of the states with existing infection prevention and control structures within the COVID emergency response, only 40 % were functional. The average percentage of pre-test and post-test scores were 60.8 ± 13.4 and 67.8 ± 9 0.3 respectively, showing a statistically significant dif-  ference (p > 0.001) in trainee knowledge. Additionally, 70 % of participants evaluated the training workshop as “satisfactory” or higher in training format, relevance for daily clinical work, active participation, learning new concepts, and logistics. | Nationwide infection prevention and control training is feasible during a national  health crisis. Infection prevention and control is cardinal in the containment of epidemic-prone  diseases like COVID-19 and is invaluable in the prevention of healthcare-associated infections  in healthcare settings. |
| 55 | Sheri et al. (2018) | A scoping review of mentor training programs in medicine between 1990 and 2017 | Systemic Review Meta-Analysis | Systematic review from 1990-2017 on mentor training programmes in Medicine. | To promote effective and consistent use of mentor training in medical education, this scoping review asks what mentor training programs are available in undergraduate and postgraduate medicine and how they may inform the creation of an evidenced-based framework for mentor training.  Six reviewers adopted Arksey and O’Malley’s approach to scoping reviews to study prevailing mentor-training programs and guidelines in postgraduate education programs and in medical schools. The focus was on novice mentoring approaches. Six reviewers carried out independent searches with similar inclusion/exclusion criteria using PubMed, ERIC, EMBASE, SCOPUS, Google Scholar, and grey literature databases. Included were theses and book chapters published in English or had English translations published between 1 January 1990 and 31 December 2017. Braun and Clarke’s approach to thematic analysis was adopted to circumnavigate mentoring’s and mentor training’s evolving, context-specific, goal-sensitive, learner-, tutor- and relationally dependent nature that prevents simple comparisons of mentor training across different settings and mentee and mentor populations. | NIL | In total, 3585 abstracts were retrieved, 232 full-text articles were reviewed, 68 articles were  included and four themes were identified including the structure, content, outcomes and  evaluation of mentor training program. | Themes of structure, content, outcomes, and evaluation of mentor training programmes should provide the basis for an evidence-based, practice-guided framework for a longitudinal mentor training program in medicine and identifies the essential topics to  be covered in mentor training programs. |
| 56 | Sonntag et al. (2020) | Train the trainer course for general practice trainers in ambulatory care: the Berlin model | Feasibility study | Train-the-trainer seminar developed for didactic competencies of general practice trainers.  N=46 | Demands for a stronger competence orientation of specialty postgraduate medical training require the expansion of the didactic qualifications of those responsible for postgraduate medical training. In the context of the foundation of the Berlin competence center for postgraduate general practice training, a train the trainer basic seminar was designed together with the Berlin chamber of physicians. The seminar aims to convey formal-legal aspects in close connection with the development of didactic competences of the general practice trainers. This article presents the didactic concept, focal points and the schedule of the one-and-a-half-day seminar to be able to adapt it to one's own context.  After the seminars, participants filled out an evaluation form. The questionnaire included the subjective experiences of increased competence, the relevance of the contents, and the satisfaction with the structure and methods of the seminar. The data were analyzed descriptively. | One-and-a-half day seminar | Clarification of questions regarding further training was achieved, with didactic skills further enhanced.  Since June 2018, 46 general practice trainers have participated in one of three train the trainer seminars. 97.6% of the participants were very satisfied or satisfied with the overall seminar and felt that the timeframe was right, 92.7% would recommend the seminar to colleagues. 68.3% fully agreed that by attending the seminar they were able to improve their didactic skills, 90% were confident that they could integrate what they had learned into their work as general practice trainers. 85.4% stated that they had reflected on their role as trainers.In particular, the atmosphere, the high degree of interactivity and the protected framework for collegial exchange were positively emphasized. | Successful design of a train the trainer seminar which, on the one hand, met the needs of the general practice trainers for clarification of formal-legal questions of their further training activities and, on the other hand, allowed the further development of didactic skills. At the moment, a modular program is being planned in order to give general practice trainers the opportunity to expand their didactic competence and regularly exchange ideas with colleagues. |
| 57 | Sood et al. (2016) | Mentoring Early-Career Faculty Researchers Is Important—But First “Train the Trainer” | Qualitative | Presentation of a framework for mentor development | To address the scarcity of skilled  research mentors and the relative lack  of attention to and recognition of the  importance of a supportive institutional  climate for mentoring, this paper aims to develop programs to “train the trainer” as well as programs and policies to support  mentors. | NIL | In this Commentary, the authors offer a  comprehensive two-pronged framework for mentor development with elements  that address both individual mentoring competencies and the institutional climate for mentoring. The framework  depicts the gaps, activities, and outcomes that a mentor development program can address. Activities directed  at changing the institutional climate related to mentor development should  complement training activities for  individual mentors. | The authors propose  that employing this framework’s  approach to mentor development  will lead to the desired impact: to increase the competence, productivity,  and retention of a diverse clinical and translational research workforce. |
| 58 | Sorkness et al. (2013) | Research Mentor Training: Initiatives of the University of Wisconsin Institute for Clinical and Translational Research | Qualitative and Quantitative | Mentor training programme at the University of Wisconsin-Madison | The University of Wisconsin-Madison (UW) has served as a hub of multidisciplinary expertise in both research mentor and mentee training and evaluation, with long-standing, federally funded efforts to support innovative practice, training interventions, and research to improve training programs for diverse scholars in science, technology, engineering, mathematics, and medicine (STEMM). Ten years ago, the Wisconsin Program for Scientific Teaching, codirected by Drs. Pfund, Miller, and Handelsman, led an effort to train future biology faculty to become more effective research mentors. Cohorts of biology graduate students, postdoctoral trainees, faculty, and staff met to discuss mentoring challenges and solutions, generating case studies and discussion questions along the way. | 4 2-hour sessions | Published evaluations of the Entering Mentoring seminars indicate that mentors who participate in training gain important skills. These trained mentors are more likely to consider issues of diversity, discuss expectations with their mentees, and to seek the advice of their peers. | Mentor training resulted in the acquisition of teaching skills, along with improving mentoring capacity |
| 59 | Sorkness et al. (2017) | A new approach to mentoring for research careers: the National Research Mentoring Network | Qualitative | Describing the structure and activities of the National Research Mentoring Network | Effective mentorship is critical to the success of early stage investigators, and has been linked to enhanced mentee productivity, self-efficacy, and career satisfaction. The mission of the National Research Mentoring Network (NRMN) is to provide all trainees across the biomedical, behavioral, clinical, and social sciences with evidence-based mentorship and professional development programming that emphasizes the benefits and challenges of diversity, inclusivity, and culture within mentoring relationships, and more broadly the research workforce. The purpose of this paper is to describe the structure and activities of NRMN. | Various interventions across the network, including online and in-person trainings | NRMN serves as a national training hub for mentors and mentees striving to improve their relationships by better aligning expectations, promoting professional development, maintaining effective communication, addressing equity and inclusion, assessing understanding, fostering independence, and cultivating ethical behavior. Training is offered in-person at institutions, regional training, or national meetings, as well as via synchronous and asynchronous platforms; the growing training demand is being met by a cadre of NRMN Master Facilitators. NRMN offers career stage-focused coaching models for grant writing, and other professional development programs. NRMN partners with diverse stakeholders from the NIH-sponsored Diversity Program Consortium (DPC), as well as organizations outside the DPC to work synergistically towards common diversity goals. NRMN offers a virtual portal to the Network and all NRMN program offerings for mentees and mentors across career development stages. NRMNet provides access to a wide array of mentoring experiences and resources including MyNRMN, Guided Virtual Mentorship Program, news, training calendar, videos, and workshops. National scale and sustainability are being addressed by NRMN “Coaches- in-Training” offerings for more senior researchers to implement coaching models across the nation. “Shark Tanks” provide intensive review and coaching for early career health disparities investigators, focusing on grant writing for graduate students, postdoctoral trainees, and junior faculty. | Mentors better understand mentoring models, roles and responsibilities, structure and dynamics, along with methods to facilitate effective mentoring. |
| 60 | Tillman et al. (2012) | Policies, Activities, and Structures Supporting Research Mentoring: A National Survey of Academic Health Centers With Clinical and Translational Science Awards | Qualitative | Review of policies and activities supporting mentoring at institutions receiving Clinical and Translational Science Awards | This article aims to document the frequency of policies and activities in support of mentoring practices at institutions receiving a U.S. National Institutes of Health’s Clinical and Translational Science Award (CTSA).  The study consisted of a 69-item survey with questions about the inclusion (formal or informal) of policies, activities, and structures supporting mentoring within CTSA-sponsored research (i.e., KL2 programs) and, more broadly, in the CTSA’s home institution. The survey, conducted from November 2010 through January 2011, was sent to the 55 institutions awarded CTSAs at the time of the survey. Follow-up phone interviews were conducted to clarify responses as needed. | NIL | Fifty-one of 55 (92%) institutions completed the survey for institutional programs and 53 of 55 (96%) for KL2 programs. Responses regarding policies and activities involving mentor criteria, mentor–mentee relationship, incentives, and evaluative mechanisms revealed considerable variability between KL2 and institutional programs in some areas, such as having mentor qualification criteria and processes to evaluate mentors. The survey also identified areas, such as training and women and minority mentoring programs, where there was frequent sharing of activities between the institutional and KL2 programs.  Varying policies and activities are used to increase qualifications of mentors, along with optimising the mentor-mentee relationship, incentives, and evaluation methods. | KL2 programs and institutional programs tend to have different preferences for policies versus activities to optimize qualification of mentors, the mentor–mentee relationship, incentives, and evaluation mechanisms. Frequently, these elements are informal. Individuals in charge of implementing and maintaining mentoring initiatives can use the results of the study to consider their current mentoring policies, structures, and activities by comparing them with national patterns within CTSA institutions. |
| 61 | Tsen et al. (2012) | The Development, Implementation, and Assessment of an Innovative Faculty Mentoring Leadership Program | Qualitative | Faculty Mentoring Leadership Programme to improve mentoring and leadership skills.  N=16 | In response to a faculty survey on mentoring, leaders at Brigham and  Women’s Hospital developed the Faculty Mentoring Leadership Program (FMLP) as a peer-learning  experience for mid-career and senior faculty physician and scientist mentors to enhance  their skills and leadership in mentoring and create a supportive community of mentors. A planning  group representing key administrative, educational, clinical, and research mentorship  constituencies designed the nine-month course.  Participants met monthly for an hour and a half during lunchtime. Two co-facilitators engaged the  diverse group of 16 participants in interactive discussions about cases based on the participants’  experiences. While the co-facilitators discussed with the participants the dyadic mentor-mentee relationship, they specifically emphasized the value of engaging multiple mentors and establishing mentoring networks. | Monthly 1.5 hour case-based discussion sessions | In response to post-session and post-course (both immediately and after six  months) self-assessments, participants reported substantive gains in their mentoring confidence and effectiveness, experienced a renewed sense of enthusiasm for mentoring, and took initial steps  to build a diverse network of mentoring relationships. | Effective mentoring is an important component of academic success, but few programs exist  to both improve the effectiveness of established mentors and cultivate a mentoring  community. Using an innovations framework, this article presented the rationale, design,  implementation, evaluation, and ongoing impact of the Faculty Mentoring Leadership  Program (FMLP) at Brigham and Women’s Hospital (BWH). |
| 62 | Varma et al. (2016) | Experience of a faculty development workshop in mentoring at an Indian medical college | Qualitative | Mentoring workshop for mentors of undergraduate medical students  N=28 | The academic leadership of the college identified the need to  change the focus of their mentoring programme for undergraduates from mere problem-solving to professional and personal development of mentees.  A core group of faculty designed and implemented a workshop on mentoring for 28 mentors. The workshop  included reflections on the participants’ previous experiences about mentoring, discussion on perceptions of mentees about the existing mentoring programme, self-analysis of mentoring skills, overview of the Surrendering, Accepting, Gifting and Extending  (SAGE) model and demonstration of effective mentoring skills using role plays and a film. We collected written anonymous feedback from participants at the end of the workshop to elicit their responses regarding various aspects of the programme, change in their views about mentoring and suggestions for future workshops. | Workshop regarding Surrendering, Accepting, Gifting and Extending model | A majority of the participants (17, 60.7%) said  that role plays and reflection on role plays were the most  valuable part of workshop as they provided clarity on the  concepts about mentoring. The most frequently identified take-home messages were: building trust with the mentee (7,  25%), balance in life and approach towards the mentee (6, 21.4%), and understanding that mentoring is a process geared towards personal and professional development of the  mentee (6, 21.4%). | The participants’ reaction to the workshop  was positive. The responses of participants suggested that the  workshop was successful in changing their views regarding the purpose of the mentoring programme. |
| 63 | Vlachou et al. (2022) | The development and evaluation of “Training the trainer” curriculum for surgical residents: Feasibility study | Review | Training of technical surgical skills in the context of cascade training.  N=41 | The aim of this proof-of-concept study was to design a bespoke TTT curriculum for surgical  technical skills and evaluate its impact.  A bespoke TTT curriculum was developed to address key teaching surgical skills including a  training framework, and performance enhancing feedback. The curriculum was delivered to 41 junior surgical residents in this feasibility study and focused on promoting a training framework including three domains; “set”  involving pre-operative preparation, “dialogue” referring to teaching techniques and “closure” covering structured feedback. It was evaluated using Kirkpatrick’s model: (i) course feedback; (ii) training quality assessment  on a suturing simulated scenario using (a) Competency Assessment Tool (CAT) and a (b) Structured Training Trainer Assessment Report (STTAR) tool; (iii-iv) follow-up survey after one year. | 2-hour interactive workshop | The TTT curriculum was well-perceived, with a median score of 4/5 (“agree”) across all components of  evaluation forms. The simulated training scenario demonstrated a significant reduction in suturing errors  following delivery of training (pre-TTT [4.25; IQR:4.42]; post-TTT [2.34; IQR:2.38], p-value = 0.014).  Improvement in teaching was also noted and reflected in ‘Set’ (pre-TTT [3.50; IQR: 3.00] and post-TTT [5.00;  IQR: 0.00], p-value = 0.019) and ‘Closure’ (pre-TTT [4.75; IQR: 1.88] and post-TTT [5.00; IQR: 0.00], p value =  0.007). 25% of participants contributed to the long-term survey highlighting that most practiced skills within 6  months of the curriculum with positive feedback from their learners.  Overall, mentors made fewer errors post training, along with improvements in teaching ability. Positive feedback from mentees. | This proof-of-concept study confirms the feasibility and acceptability of delivering a bespoke Train-  The-Trainer curriculum to surgical residents. It provides a structured training framework that can enhance  teaching technical skills. |
| 64 | Wahab et al. (2016) | Creating Effective Interprofessional Mentoring Relationships in Palliative Care - Lessons from Medicine, Nursing, Surgery and Social Work | Review | Review on Operationalization of mentoring programme in palliative care | To address the lack of data on operationalizing an interprofessional mentoring program in Palliative Care, this paper scrutinized mentoring approaches in medicine, surgery, nursing and medical social work to  identify common elements of mentoring within their respective practices that will provide the basis of an interprofessional mentoring in Palliative Care. | NIL | Thematic analysis of 20 reviews of undergraduate and postgraduate mentoring programs in medicine, surgery and nursing suggest that successful mentoring programs are underscored by effective nurturing and support of mentoring relationships. Successful mentoring relationships are built on strong relational ties between mentees and mentors.  Mentors are selected for certain characteristics. Effective mentoring provides various personal and professional benefits. | Delineating the key elements to effective mentoring relationships  allow for the forwarding of a basic framework to enhance relational ties within interdisciplinary mentoring in Palliative Care and the proffering of an evidence-based platform for the adoption of a cognitive apprenticeship model that can  guide the operationalization of a multiprofessional mentoring program in Palliative Care. |
| 65 | Walters et al. (2016) | Mentoring the Mentors of Underrepresented Racial/Ethnic Minorities Who are Conducting HIV Research: Beyond Cultural  Competency | Review | Review on HIV researcher mentor training programmes, particularly on diversity training | The majority of literature on mentoring focuses on mentee training needs, with significantly less guidance for the mentors. Moreover, many mentoring the mentor models assume generic (i.e. White) mentees with little attention to the concerns of underrepresented racial/ethnic minorities (UREM). This has led to calls for increased attention to diversity in research training programs, especially in the field of HIV where racial/ethnic disparities are striking. Diversity training tends to address the mentees' cultural competency in conducting research with diverse populations, and often neglects the training needs of mentors in working with diverse mentees.Thus, this article aims to critique the framing of diversity as the problem (rather than the lack of mentor consciousness and skills), highlight the need to extend mentor training beyond aspirations of cultural competency toward cultural humility and cultural safety, and consider challenges to effective mentoring of UREM, both for White and UREM mentors. | NIL | Mentor training must achieve goals of cultural humility and safety, beyond those of cultural competency. | There is a need to diversify the research workforce to address health disparities. Mentors have diversity training needs that have to be addressed. |
| 66 | Welch et al. (2012) | The Women in Emergency Medicine Mentoring Program: An Innovative Approach to Mentoring | Qualitative | Mentoring programme for women in Emergency Medicine  N=72 | This study aims to describe the content, perceived value, and  ongoing achievements of a mentoring program for  women in emergency medicine.  The program offered mentoring for female faculty and residents in an academic emergency medicine department. Volunteers participated in group mentoring sessions using a mosaic of vertical and peer mentoring. Sessions focused on topics specific to  women in medicine. An anonymous, electronic survey was sent to women who participated during 2004–2010 to assess the perceived value of the program and to collect qualitative feedback. Preliminary achievements fulfilling the program’s goals were  tracked. | 2-hour sessions as biannual gatherings | Allows for the expansion of female mentoring pool, creating a supportive environment among other benefits. | This innovative model for mentoring women is perceived as a valuable asset to the academic department and residency. It offers the unique combination of  expanding a female mentor pool by recruiting alumni and using a mosaic of vertical and peer mentoring. |
| 67 | Wong et al. (2017) | Faculty–Resident “Co-learning”: A Longitudinal Exploration of an Innovative Model for Faculty Development in Quality Improvement | Qualitative | Effectiveness of co-learning of mentors and mentees for Quality Improvement  N=29 | To examine the effectiveness of  co-learning, wherein faculty and  trainees learn together, as a novel  approach for building quality  improvement (QI) faculty capacity.  Hence, from July 2012 through September 2015, the authors conducted 30 semistructured interviews with 23 faculty  participants from the Co-Learning  QI Curriculum of the Department  of Medicine, Faculty of Medicine,  University of Toronto, and collected  descriptive data on faculty participation and resident evaluations of teaching  effectiveness. Interviewees were from 13 subspecialty residency programs  at their institution. | 2 workshops, as part of curriculum spanning 1 year | Of the 56 faculty participants, the  Co-Learning QI Curriculum trained 29 faculty mentors, 14 of whom taught formally. Faculty leads with an academic QI role, many of whom had prior QI training, reinforced their QI knowledge while also developing QI mentorship and teaching skills. Co-learning elements that contributed to QI teaching skills  development included seeing first how the QI content is taught, learning through project mentorship, building experience  longitudinally over time, a graded transition toward independent teaching, and a supportive program lead. Faculty with  limited QI experience reported improved QI knowledge, skills, and project facilitation but were ambivalent about assuming a  teacher role. Unplanned outcomes for both groups included QI teaching outside of the curriculum, applying QI principles to other work, networking, and strengthening one’s QI professional role.  All in all, mentors’ knowledge, alongside ability to teach and mentor on quality improvement increased. | The Co-Learning QI Curriculum was  effective in improving faculty QI  knowledge and skills and increased faculty  capacity to teach and mentor QI. Findings  suggest that a combination of curriculum  and contextual factors were critical to  realizing the curriculum’s full potential. |
| 68 | Wu et al. (2016) | Toward an Interprofessional Mentoring Program in Palliative Care - A Review of Undergraduate and Postgraduate Mentoring in Medicine, Nursing, Surgery and Social Work | Review | Review of mentoring programmes in Palliative Care | Key to effective Palliative Care is interdisciplinary collaboration and holistic support of members of the multidisciplinary team. Mentoring is increasingly seen as being a critical facet of this process however; there is a dearth of guidance on establishing such a program within the Palliative Care setting. To fill this gap, this review analyzes mentoring programs in medicine, surgery, nursing and social work in order to identify key elements and common facets of successful mentoring programs that can be used to create a multi-professional mentoring program in Palliative Care. | NIL | A total of 20 reviews were included. One review was on mentoring in medicine and nursing, 10 in medicine, 4 in surgery and 5 in nursing. There were no reviews of mentoring in social work. Thematic analysis revealed 3 themes, which were definition of mentoring, components of a mentoring approach and elements of the mentoring process  Mentor training can be performed through workshops or seminars, books and manuals, or simulations and practical exercises. | Despite its context sensitive, goal specific and mentee- and mentor- dependent features, common features in mentoring in medicine, surgery and nursing lay the foundation for a learning theory of interprofessional mentoring that can guide construct effective mentorship programs. |
| 69 | Yin et al. (2015) | Sustaining the Clinical Translational Research Workforce: Training and Empowering the Next Generation of Investigators | Perspective | Perspective on development of Clinical Translational Research investigators | There is mounting concern that clinician scientists are a vanishing species, and that the pipeline for clinical translational research (CTR) investigators is in jeopardy. For the majority of current junior CTR investigators, the career path involves first obtaining a National Institutes of Health (NIH) funded K-type career development award, particularly K08 and K23, and subsequently an NIH R01. This transition, popularly referred to as K2R, is a major hurdle with a low success rate and gaps in funding.  In this Perspective, the authors identify factors that facilitate K2R transition and important aspects of increasing and sustaining the pipeline of CTR investigators. They also highlight significant differences in success rates of women and those underrepresented in biomedical research. | NIL | Formal mentor training is effective in ensuring success for early researchers. Early career exposure to research methodology, protected time, multidisciplinary mentoring, and institutional “culture shift” are important for fostering and rewarding team science. Mentoring is the single most important contributor to K2R success, and emerging evidence suggests that formal mentor training and team mentoring are effective.  Leadership training can empower junior investigators to thrive as independent CTR investigators. | Future research should focus on delineating the difference between essential and supplemental factors to achieve this transition, and mentoring methods that foster success, including those that promote K2R transition of women and those underrepresented in biomedical research. The Clinical Translational Science Awards National Consortium is well positioned to test existing models aimed at shortening the timeframe, increasing the rate of K2R transition, and identifying strategies that improve success. |
